# Single Application of Low-Dose, Hydroxyapatite-Bound BMP-2 or GDF-5 Induces Long-Term Bone Formation and Biomechanical Stabilization of a Bone Defect in a Senile Sheep Lumbar Osteopenia Model

**DOI:** 10.3390/biomedicines10020513

**Published:** 2022-02-21

**Authors:** Ines Hasenbein, André Sachse, Peter Hortschansky, Klaus D. Schmuck, Victoria Horbert, Christoph Anders, Thomas Lehmann, René Huber, Alexander Maslaris, Frank Layher, Christina Braun, Andreas Roth, Frank Plöger, Raimund W. Kinne

**Affiliations:** 1 Department of Orthopedics, Jena University Hospital, Waldkliniken Eisenberg GmbH, 07607 Eisenberg, Germany; i.hasenbein@web.de (I.H.); a.sachse@waldkliniken-eisenberg.de (A.S.); victoria.horbert@med.uni-jena.de (V.H.); alexander.maslaris@krupp-krankenhaus.de (A.M.); f.layher@waldkliniken-eisenberg.de (F.L.); 2 Experimental Rheumatology Unit, Department of Orthopedics, Jena University Hospital, Waldkliniken Eisenberg GmbH, 07607 Eisenberg, Germany; christina-braun@hotmail.de; 3 Leibniz Institute for Natural Product Research and Infection Biology (Leibniz-HKI), 07745 Jena, Germany; peter.hortschansky@leibniz-hki.de; 4 Johnson & Johnson Medical GmbH, DePuySynthes, 22851 Norderstedt, Germany; kschmuck@its.jnj.com; 5 Division of Motor Research, Pathophysiology and Biomechanics, Experimental Trauma Surgery, Department for Hand, Reconstructive, and Trauma Surgery, Jena University Hospital, 07743 Jena, Germany; christoph.anders@med.uni-jena.de; 6 Institute of Medical Statistics, Computer Sciences and Data Sciences, Jena University Hospital, 07743 Jena, Germany; thomas.lehmann@med.uni-jena.de; 7 Institute of Clinical Chemistry, Hannover Medical School, 30625 Hannover, Germany; huber.rene@mh-hannover.de; 8 Bereich Endoprothetik/Orthopädie, Klinik für Orthopädie, Unfallchirurgie und Plastische Chirurgie, Uniklinik Leipzig AöR, 04103 Leipzig, Germany; andreas.roth@medizin.uni-leipzig.de; 9 BIOPHARM GmbH, Czernyring 22, 69115 Heidelberg, Germany; frank.ploeger@gmx.de

**Keywords:** animal model, sheep, osteopenia, growth factors, osteoinductive, BMP-2, GDF-5

## Abstract

Effects of hydroxyapatite (HA) particles with bone morphogenetic BMP-2 or GDF-5 were compared in sheep lumbar osteopenia; in vitro release in phosphate-buffered saline (PBS) or sheep serum was assessed by ELISA. Lumbar (L) vertebral bone defects (Ø 3.5 mm) were generated in aged, osteopenic female sheep (*n* = 72; 9.00 ± 0.11 years; mean ± SEM). Treatment was: (a) HA particles (2.5 mg; L5); or (b) particles coated with BMP-2 (1 µg; 10 µg) or GDF-5 (5 µg; 50 µg; L4; all groups *n* = 6). Untouched vertebrae (L3) served as controls. Three and nine months post-therapy, bone formation was assessed by osteodensitometry, histomorphometry, and biomechanical testing. Cumulative 14-day BMP release was high in serum (76–100%), but max. 1.4% in PBS. In vivo induction of bone formation by HA particles with either growth factor was shown by: (i) significantly increased bone volume, trabecular and cortical thickness (overall increase HA + BMP vs. control close to the injection channel 71%, 110%, and 37%, respectively); (ii) partial significant effects for bone mineral density, bone formation, and compressive strength (increase 17%; 9 months; GDF-5). Treatment effects were not dose-dependent. Combined HA and BMPs (single low-dose) highly augment long-term bone formation and biomechanical stabilization in sheep lumbar osteopenia. Thus, carrier-bound BMP doses 20,000-fold to 1000-fold lower than previously applied appear suitable for spinal fusion/bone regeneration and improved treatment safety.

## 1. Introduction

Osteoporosis is a systemic skeletal disease characterized by high individual disease load and severely impaired quality of life, causing major socioeconomic health problems worldwide [1]. The reduced bone mass and disturbed microarchitecture result in loss of mechanical strength and increased fracture risk, leading to fractures of lumbar spine, radius, proximal femur, and other parts of the lower extremity [2]. Therapeutic options for acute vertebral body fractures include well-established surgical procedures such as spondylodesis, but minimally invasive methods (e.g., vertebroplasty and balloon kyphoplasty) with bone cements are increasingly used, due to rapid and substantial reduction of pain and clear improvement of lumbar biomechanics in 90% of the patients [3,4].

In animal studies, bone morphogenetic proteins (BMPs) and growth/differentiation factors (GDFs) have been used for the surgical filling of traumatic or tumor non-loaded bone defects [5,6,7] and the spinal fusion of acute vertebral body fractures [8,9]. rhBMP2 (InFUSE^®^; Amplify^®^) is licensed under a premarket approval (PMA) for marketing and clinical use in the United States and has been developed as a product for the repair of long bone non-union fractures and lumbar spinal fusion in humans ([10] and references therein). However, clinically approved high-dose BMP application (3.5 to 12 mg; [11,12] and references therein) results in an initial burst release believed to be responsible for inflammatory and osteoclastic side effects, and may contribute to ectopic bone formation, potential risk of osteosarcoma and autoantibody formation, or spinal complications [13,14,15]. To reduce these side effects, substantially smaller BMP doses (present study) and/or a modification of this initial release by alterations of the carrier material may be advantageous [16,17,18,19].

The main osteogenic growth factors BMP-2, BMP-4, BMP-6, BMP-7, and GDF-5 (alias BMP-14) are members of a large family of 40 multifunctional proteins [20,21,22]. In adult vertebrates, BMPs act as differentiation proteins and mitogens, and have central importance for the induction of bone and cartilage formation [22,23,24,25]. BMPs induce de novo bone synthesis or regeneration of fractured bone via the activation of pluripotent stem cells, mesenchymal progenitor cells, and/or resident osteoblasts [22]. Compared to BMP-2, GDF-5 has been described as a growth factor with lower osteoinductive potential in vivo [26]; this may be mainly caused by different receptor profiles of the target cells [8], which cause the activation of different signaling pathways [27].

Due to their short half-life and the risk of side effects (autoantibody induction, ectopic bone formation, potential placental passage, theoretical induction of osteosarcoma) [13,14,15], BMPs cannot be systemically injected but must be used locally and in a carrier-associated form. The combination with a carrier leads to delayed release of BMP at the application site and allows the use of lower BMP doses [11,12,28,29,30]. Current carrier matrices are components of the extracellular matrix ([8,31,32,33]), ceramics (e.g., hydroxyapatite (HA), tricalcium phosphate; [34,35]), and synthetic polymers [36,37], some of which are osteoconductive [38]. To be accepted as a bone graft biomaterial, the carrier matrix must be highly biocompatible and free of any toxicity, immunogenicity, or carcinogenicity [13,36].

In the present study, recombinant human BMP-2 and GDF-5 were used, both of which can be produced with high yield and low cost in *Escherichia coli* [39]. BMP doses were pre-determined in cell culture experiments with the C2C12 or ATDC-5 cell lines, respectively. The biocompatible carrier HA was then coated with BMP-2 or GDF-5, applied as particle suspension to the lumbar vertebra L4 of aged, osteopenic sheep [40], and comparatively analyzed for their therapeutic effects. In addition, the in vitro release of BMP-2 and GDF-5 from the HA was analyzed after incubation in phosphate-buffered saline (PBS) or sheep serum.

The present study shows that combined delivery of HA and low-dose BMP-2 (1 µg) or GDF-5 (5 µg) is highly suitable for the therapy of lumbar osteopenia in aged sheep [40], including long-term augmentation of bone structure and bone formation, as well as biomechanical stabilization.

## 2. Materials and Methods

### 2.1. Production of Recombinant Human BMP-2 and GDF-5 in E. coli

The biotechnological production of non-glycosylated BMP-2 was accomplished using established and patented procedures (patent DE 199 44 626 A1; [41,42]) also commonly applied for clinical trials and therapy in humans (e.g., https://clinicaltrials.gov; accessed on 15 February 2022; ClinicalTrials.gov identifiers: NCT00813813, NCT01158924, NCT01182337, NCT01124006), that of GDF-5 by analogous procedures. The material was analyzed for endotoxin and total DNA content by a commercial partner using established procedures (Limulus Amebocyte Lysate test; [43]). The results revealed very low contamination for endotoxin (BMP-2: endotoxin < 0.11 IU/mg; GDF-5: endotoxin < 0.17 IU/mg) and DNA content (BMP-2: DNA content < 0.01 pg/µg), both well below the previously accepted threshold values for therapeutic products (https://www.fda.gov/media/83477/download (accessed on 15 February 2022); [44,45]).

### 2.2. In Vitro Evaluation of the Dosages for BMP-2 and GDF-5 Combined with Bone Graft Substitutes (HA) for Application in Animal Experiments

According to the supplier, the pulverized carrier material (100% pure ceramic HA; particle size of 29 ± 1 µm; Camceram III HA coating powder™; Cam Implants BV, Leiden, The Netherlands) was produced by spray drying and reached a density close to the theoretical maximum. The HA particles were coated with the two different growth factors in six different quantities (0 to 189.00 µg BMP-2/50 mg HA; 0 to 197.55 µg GDF-5/50 mg HA; for details, see Section 2.3. below). The transformed, pre-myoblastic C2C12 cell line (ATCC-Nummer: CRL-1772) was selected to assess the bioactivity of BMP-2 released from the carrier, while the teratocarcinoma-derived chondrogenic cell line ATDC-5 (Riken BRC cell bank, RCB0565) was used for GDF-5 [46,47]. These two cell lines were used due to their differential receptor expression, i.e., C2C12 cells only react to stimulation with BMP-2 (but not to GDF-5), whereas ATDC-5 cells do react to stimulation with GDF-5 [48,49].

In the case of C2C12 (for BMP-2), Dulbecco’s modified Eagle’s medium (DMEM™; Sigma Aldrich, Taufkirchen, Germany) with 1.2 g/L sodium bicarbonate (Merck, Darmstadt, Germany) and 5% fetal calf serum (Gibco™, Invitrogen, Karlsruhe, Germany) was used, in the case of ATDC-5 (for GDF-5) DMEM nutrient mixture F-12 HAM (DMEM™/F-12, Sigma Aldrich) with 3.7 g/L sodium bicarbonate (Merck) and 10% fetal calf serum (Sigma Aldrich). Both cell lines were cultured in 6-well plates (Greiner Bio-One, Frickenhausen, Germany) at a density of 100,000 cells per well. After adherence for 24 h, the cells were covered with the growth-factor-coated HA particles to simulate their direct contact following in vivo injection and were subsequently cultured for an additional 3 days. This culture time was chosen to avoid hypertrophic alterations of the cells, leading to constitutively increased alkaline phosphatase (ALP) activity. ALP activity, a biochemical indicator for osteoinductive growth factors, was determined in a Fluor-S™ MultiImager (Bio-Rad, Munich, Germany) using an ELISA kit (BM chemiluminescence ELISA substrate (AP)™, Cat. No. 11 759 779 001; Roche, Grenzach-Wyhlen, Germany).

Briefly, culture medium of the respective cells was collected, diluted 1:4 in a microfuge tube with dilution buffer, and incubated with a sealed cap for 30 min in a water bath at 65 °C. Samples were then collected by centrifugation in a microcentrifuge for 1 min at full speed and transferred to an ice bath (0 to +4 °C). A final concentration of 15 mM L-homoarginine (MW 224.7; 34 mg/10 mL) was added to the assay buffer to block contaminating isoforms of alkaline phosphatase, and 50 µL of heat-inactivated sample was pipetted into a microplate (black or white). After preparing the MultiImager, the reaction was started with 100 µL substrate reagent and the sample was first incubated for 10 min at room temperature (RT) under gentle rocking and then transferred to the MultiImager to integrate the chemiluminescence for 250 s.

Therapeutic in vivo doses for the animal study were derived from the in vitro evaluation as 1 µg BMP-2/2.5 mg HA (low dose; lowest dose with a detectable effect; pharmaceutical formulation 2 µg BMP-2/5 mg HA, see below; in vivo application of ½ dose) and 10 µg BMP-2/2.5 mg HA (high dose; half-maximal effect). To account for the presumed lower biological activity of GDF-5 [26], doses for GDF-5 were 5 µg (low dose; half-maximal effect; pharmaceutical formulation 10 µg GDF-5/5 mg HA) and 50 µg (high dose; saturation effect).

### 2.3. In Vitro Release of BMP-2 and GDF-5 from the HA Particles

For analysis of the in vitro release from the therapeutically applied formulations in phosphate-buffered saline (PBS; 0.15 M NaCI, 6.5 mM Na_2_HPO_4_, 1.5 mM KH_2_PO_4_, pH 7.4) or sheep serum (obtained under permission number 02-21/05; Governmental Commission for Animal Welfare, Free State of Thuringia, Berlin, Germany), the pulverized carrier material was coated with two different pharmaceutical doses of the growth factors (2 µg and 20 µg BMP-2/5 mg HA; 10 µg and 100 µg GDF-5/5 mg HA; triplicate samples for each concentration) by incubating the HA particles with a BMP-containing solution (200 µL of 50% (*v*/*v*) acetonitrile, 0.1% trifluoroacetic acid) and then drying the HA particles in a SpeedVac vacuum concentrator (Fisher Scientific GmbH, Schwerte, Germany). Based on the theoretical calculation of the surface area required for a single layer binding of all growth factor molecules, the loading protein should have been completely immobilized on the surface of the HA particles.

To analyze the in vitro BMP release, the samples were incubated at RT in Eppendorf tubes (Eppendorf SE, Hamburg, Germany) under constant agitation with 1.2 mL of PBS (pH 7.2) or sheep serum for 0.5, 1, 4, or 6 h, as well as 1, 2, 4, 7, 9, 11, and 14 days (see Table S1). For the 0 h time point, PBS or serum was analyzed without exposure to the HA particles; for later time points the particle suspensions were centrifuged, the supernatant was removed (stored at −80 °C for ELISA analysis), and 1.2 mL of fresh PBS or serum was added to the particles.

The growth factor release was quantified by ELISA (sensitivity for BMP-2: 50 to 400 pg/mL; for GDF-5: 100 to 200 pg/mL). For this purpose, the supernatants were thawed and the BMP concentrations were measured using an ELISA assay developed by the company Biopharm GmbH (Heidelberg, Germany). On the basis of their sensitivity, the ELISA systems were capable of detecting growth factor release down to 0.01% of the coated protein.

### 2.4. Animal Experiments

Aged, osteopenic, female sheep (total of 72 animals; Schwarzkopf-Merino; 6–10 years old; 9.00 ± 0.11 years; mean ± SEM; 45–76 kg body weight) were used for osteodensitometry, histological analyses, and biomechanical testing [40].

Permission for the animal experiments was obtained from the Governmental Commission for Animal Welfare (Free State of Thuringia, Germany, registration number: 02-21/05) according to §15 of the current national Animal Welfare Act. A total of 48 animals were assigned to 8 groups (*n* = 6 each) differing in the recombinant growth factors (BMP-2/GDF-5), the dosage (low and high dose), and the experimental time periods (short term/long term; Table 1). Power analyses (G*power [50]) for differences among the various therapies in the 1 µg BMP-2 groups (control/HA/HA-BMP) at 3 and 9 months for the structural parameter BV/TV confirmed that group sizes between three and five animals were sufficient to detect differences with an alpha error probability of 0.05, a power (1-ß error probability) of 0.80, and an effect size between 2.14 and 7.91. Additional 24 animals of the same age range (4 groups with *n* = 6 each) were exclusively used for biomechanical testing (Table 1; for details, see below).

#### 2.4.1. Surgical Procedure

First, the animals were positioned on their left side and the operation region of the animals was shaved and disinfected under sedation (Ketamin™; Zoetis Deutschland GmbH, Berlin, Germany; 100 mg/mL, 12 mL; 17 mg/kg body weight). Subsequently, an inhalation anesthesia was induced (Isofluran™; AbbVie, Ludwigshafen, Germany; 1.5 vol%) and, after mobilization of the paravertebral musculature, a bone marrow biopsy needle (Ø 3.5 mm ≈ 9 gauge, Somatex™-Medical Technology GmbH, Teltow, Germany) was positioned transpedicularly in the right side of the respective lumbar vertebral bodies (L4–L5) under the control of a mobile, radiological image converter (Ziehm Imaging GmbH, Nuremberg, Germany). A bone defect of approx. 20 mm depth was then generated by advancing the biopsy needle. This procedure was performed in analogy to the open vertebroplasty technique initially applied in human patients.

HA particles coated with BMP-2 or GDF-5 (applied dose 2.5 mg HA particles; 1 or 10 µg of BMP-2; 5 or 50 µg of GDF-5; suspended in 50 µL autologous serum as a physiological solvent, which in comparison to PBS or water also delayed the sedimentation of the HA particles) were then injected with a sterile pipette tip into the bone defect in the L4 vertebral body. The injection of non-coated HA particles into L5 and the untouched L3 served as controls. To avoid leakage of the HA particles, the injection channels in L4 and L5 were closed with a steel cortical fragment screw (diameter 4.5 mm, length 12 mm; DePuy Synthes, Raynham, MA, USA).

After the completion of surgery, animals were housed in separate paddocks with social contact for 1–2 weeks and were then returned to long-care paddocks for 3 (short term) or 9 months (long term).

#### 2.4.2. Excision of the Samples

The animals were sacrificed by intravenous injection of overdosed barbiturate (Pentobarbital™, Essex Pharma GmbH, Munich, Germany) and subsequent application of magnesium sulfate (MgSO_4_). The lumbar spine was removed using an oscillating bone saw, the steel cortical fragment screws were removed from the entry of the injection channels of L4 and L5, and finally the lumbar spine was stored at −20 °C. Due to its elevated head and polished metal surface, the open hexagon head screw was always easy to retrieve and remove (please compare with graphical abstract).

In addition, the ovaries were removed and subsequently fixed in neutrally buffered, 4% paraformaldehyde (PFA). This was conducted to assess the cyclic activity of the old female sheep and thus a possible post-menopausal component of the osteoporosis model. However, histological analyses of 44 representative sheep (subgroup of the initial 48 animals) showed an active ovarian cycle; 88% were ovulating (18% interestrus; 70% late interestrus) and 12% were either shortly before or after the ovulation (7% estrus; 5% post-estrus) [51].

### 2.5. Digital Osteodensitometry

Osteodensitometry was conducted using a software-guided digital bone density measuring instrument (DEXA QDR 4500 Elite™; Hologic, Waltham, MA, USA) [52,53]. In order to allow an artifact-free osteodensitometry, the lumbar spine was sawn into individual vertebrae (L3 = control, L4, and L5) using an industrial wet sawing procedure; the spinous and transverse processes, as well as the covering and base plates, were removed (final height in each case 15 mm). The central, spongious area of the vertebral bodies was then imaged in the longitudinal direction, which represents the main loading axis in humans (compare with Section 2.8. below). A central rectangular region of interest in a transversal orientation with a defined size of 9 × 11 mm served as a measuring field. Osteodensitometric measurements were therefore limited to the central, spongious area of the vertebral body.

### 2.6. Histology/Histomorphometry

Quantitative static histomorphometry of the lumbar vertebral bodies (L3–L5) was performed according to published procedures [40,54,55,56,57,58,59]. After cutting the lumbar vertebral bodies into two opposite complementary parts directly at the injection channel, the analyses were carried out using two different types of histological sections (*n* = 1 each per animal and vertebral body): (i) decalcified paraffin sections (7 μm thickness stained by hematoxylin-eosin; [60]) or (ii) plastic-embedded sections stained with trichrome stain according to Masson-Goldner [61] after fixation in Carnoy’s solution for 12 h at 21 °C, dehydration in ascending alcohol series without demineralization, impregnation with a mixture of alcohol/Technovit 7200VLC = 1:1, followed by infiltration with pure Technovit 7200VLC (Heraeus Kulzer, Hanau, Germany), and embedding into methacrylate-based resin [62]. Sections were sawed to a thickness of approx. 200 μm and then ground to 5–10 μm.

Histomorphometrical parameters were analyzed in 25 randomly selected fields of each individual vertebral body in both different spongiosa localizations (immediate vicinity of the injection channel; remote area in the vertebral body center) using a standard microscope (Axiovert 200 M™, Carl Zeiss, Microimaging GmbH, Oberkochen, Germany), 400-fold magnification, a Merz counting net with 36 hits (inflection points of the sinus curves), and the corresponding software (Axioversion 3.1, Carl Zeiss, Microimaging GmbH, Oberkochen, Germany).

The following parameters were calculated according to the recommendations of the International Committee of the American Society for Bone and Mineral Research (ASBMR) [54,55,56,57]: The **structural bone parameters** bone volume/total volume (BV/TV), trabecular thickness (Tb.Th; both in *n* = 1 decalcified paraffin section); the **bone formation parameters** osteoblast surface (Ob.S/BS) and osteoid surface (OS/BS; both in *n* = 1 plastic-embedded section); and the **bone erosion parameter** eroded surface (ES/BS; in *n* = 1 decalcified paraffin section). Detailed information on the calculation of the parameters can be found in the Supplementary data (including Figure S1).

In addition, the average cortical thickness (Cort. Th; *n* = 1 decalcified paraffin section) was measured in the ventral (close to the tip of the injection channel) and right region (25 fields each) of each vertebral body.

Individual values for the different visual fields for each parameter were then averaged to obtain one mean value for each parameter, vertebral body, and animal for subsequent statistical analyses.

### 2.7. Dynamic Histomorphometrical Measurements

To determine dynamic histomorphometric indices, sheep were given intramuscular injections of oxytretracycline (OTC; 20 mg/kg body weight and 10 mg lidocaine) 1 and 2 days after surgery and subsequently at equal 3 months distance (until 6 months). Short-term animals thus received a total of three OTC injections, long-term animals instead a total of four injections.

Dynamic histomorphometrical measurements were performed in *n* = 1 plastic-embedded section using a fluorescence microscope (Axiovert 200 M™, Carl Zeiss, Microimaging GmbH, Oberkochen, Germany), 20-fold magnification, a Zeiss Plan Neofluar objective at an excitation wavelength of 390 nm and an emission wavelength of 512 nm with an AxioCam color camera (12 V DC, 0.7 A, Carl Zeiss), and the corresponding software (AxioVision 3.8, Carl Zeiss, Microimaging GmbH, Oberkochen, Germany). For image analysis, the Vidost tetra software was used (Videoplan, 1991, Carl Zeiss, Microimaging GmbH, Oberkochen, Germany). Before analysis, the 2-point (2P) distance was calibrated to 500 µm. All measured and calculated parameters are based on published nomenclature (see Supplementary data; [54,55,56,57]).

**Figure 1:**
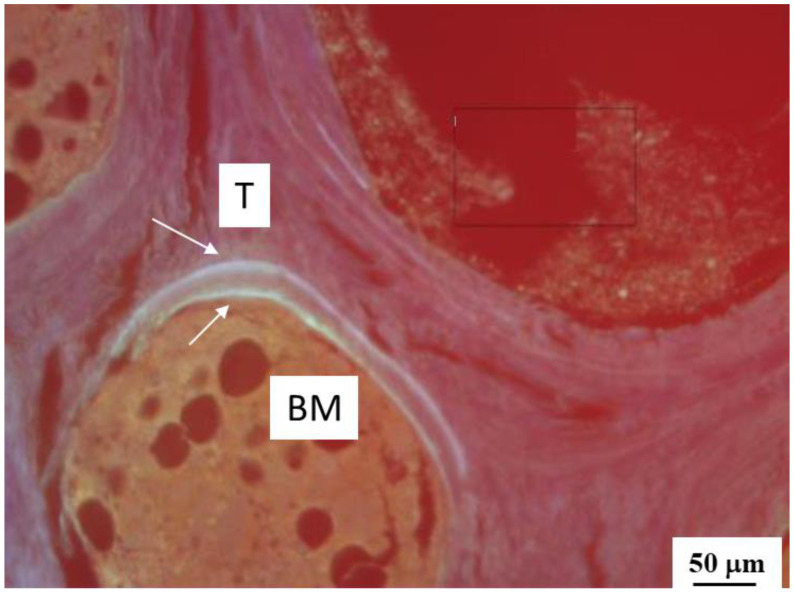
Representative image of OTC fluorescence double lines (arrows) at the interface between a bone trabecula (T) and the bone marrow (BM) close to the injection channel for the long-term, high-dose BMP-2 group.

In total, samples of 38 sheep were available for dynamic histomorphometric measurements. In every sample, 10 randomly selected visual fields in the immediate vicinity of the injection channel were examined with respect to OTC single or double lines (representative image for the long-term, high-dose BMP-2 group close to the injection channel shown in Figure 1). The total bone surface (BS) and the mineralized fraction of the BS labeled with single or double line OTC were determined (MS) and the results were expressed as the mineralized fraction (%; see below). In addition, the average distance between OTC double lines was determined over their total length (Ir.L.TH = interlabel thickness) and divided by the number of days between the subsequent injections of OTC (Ir.l.t. = interlabel time). Subsequently, the **bone formation parameters** mineralizing surface per bone surface (MS/BS), mineral apposition rate (MAR), and bone formation rate surface-based BFR/BS were calculated (see Supplementary data).

As for static histomorphometry, the individual values for each parameter in the different visual fields were then averaged to obtain one mean value for each parameter, vertebral body, and animal for subsequent statistical analyses. All evaluations were performed without considering the experimental status of the samples.

### 2.8. Biomechanical Testing (Compressive Strength)

Biomechanical compressive strength measurements were conducted as previously published [40] using a universal material testing machine (FPG 7/20-010™, Kögel, Leipzig, Germany) and the corresponding software (FRK Quicktest 2004.01™, Kögel, Leipzig, Germany). Briefly, frozen cancellous bone cylinders (10 mm diameter × 15 mm height) were harvested from the central part of the vertebral bodies containing the injection channel [63,64] using a surgical diamond hollow milling cutter (10 mm diameter; see Section 3.3 and Section 3.7 below). After a thawing time of exactly 30 min, samples were semiconfined in two semilunar clamps (minimal inner diameter of 10.1 mm and length of 9.8 mm) and then compressed along their longitudinal axis until fracturing. This axis has previously been applied with success in both animal studies and studies with human vertebrae [65,66,67] and was chosen because it represents the main loading axis in humans and is therefore of major interest for future clinical application. The applied load (loading rate 1 mm/min in air at room temperature) was recorded in a stress-strain curve until failure and the resulting force was then divided by the surface area of the specimen to obtain the compressive strength in MPa.

### 2.9. Statistical Methods

Statistical analysis was performed by using IBM^®^ SPSS^®^ 26 for Windows (SPSS Inc., IBM, Corp., Armonk, NY, USA) and R 4.1.0 [68]. All results were expressed as means ± SEM.

A linear mixed effects model was fitted to compare the effects of treatment (group), time point (time), BMP dose (dose), and distance from the injection channel (area), as well as the interactions (*) between group on one hand and the parameters time, dose, and area on the other hand, on the nine different static and dynamic histomorphometrical parameters (compare with Table S2). In this analysis, group, time, dose, and area were modeled as fixed effects with a random intercept per sheep, in order to account for multiple measurements per sheep and the correlation of these observations. Estimated means ± SEM were reported. Partial eta squared (η_p_^2^) from the model was used to assess the effect size (classification: 0.01 ≤ η_p_^2^ < 0.06 small effect size; 0.06 ≤ η_p_^2^ < 0.14 medium effect size; η_p_^2^ ≥ 0.14 large effect size). In addition, an explorative analysis was performed using the non-parametric multigroup Kruskal–Wallis and two-sided Wilcoxon tests for the comparison of paired samples within one group, and the multigroup Friedman and two-sided Mann-Whitney U-tests for statistical evaluation of differences between groups.

In selected cases with a limited number of different groups (direct comparison of BMP-2 and GDF-5 for the BV/TV or, alternatively, the data for the compressive strength), the two-sided non-parametric Wilcoxon test was used exclusively to compare paired samples in one group, the two-sided non-parametric Mann-Whitney U-test for statistical evaluation of differences between groups. In all cases, significance was accepted at *p* ≤ 0.05.

## 3. Results

### 3.1. In Vitro Evaluation of Therapeutic Dosages for BMP-2 and GDF-5

**Figure 2:**
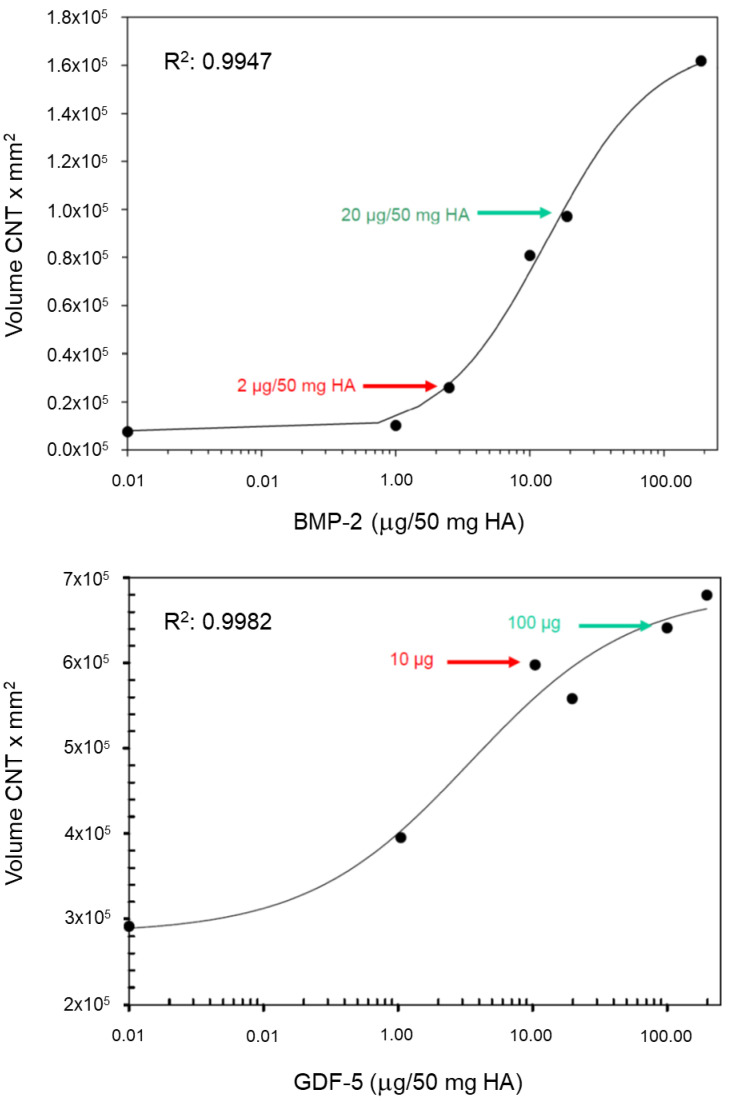
In vitro evaluation of the dosages for BMP-2 (C2C12 cell line; upper panel) and GDF-5 (ATDC-5 cell line; lower panel) for application in animal experiments by direct coculture with the growth-factor-coated HA particles for 3 days and subsequent determination of their alkaline phosphatase (ALP) activity (*n* = 3–4 experiments each; one representative result is shown; y axes show the raw values of chemiluminescent reading). Derived therapeutic doses were 1 µg BMP-2/2.5 mg HA (low dose, lowest dose with a detectable effect; pharmaceutical formulation 2 µg BMP-2/5 mg HA; in vivo application of ½ dose) or 10 µg BMP-2/2.5 mg HA (high dose; half-maximal effect). Due to the presumably lower biological activity of GDF-5, doses for GDF-5 were 5 µg (low dose; half-maximal effect) and 50 µg (high dose; saturation effect).

Therapeutic in vivo doses, as derived from the in vitro coculture of growth-factor-coated HA particles with bioindicator cells (see Methods), were defined as 5 µg GDF-5/2.5 mg HA (low dose; formulation 10 µg GDF-5/5 mg HA) and 50 µg GDF-5/2.5 mg HA (high dose; Figure 2). To account for the presumed higher biological activity of BMP-2, the respective doses were defined as 1 µg BMP-2 (low dose) and 10 µg BMP-2 (high dose).

### 3.2. In Vitro Release of BMP-2 and GDF-5 from the HA Particles

**Figure 3:**
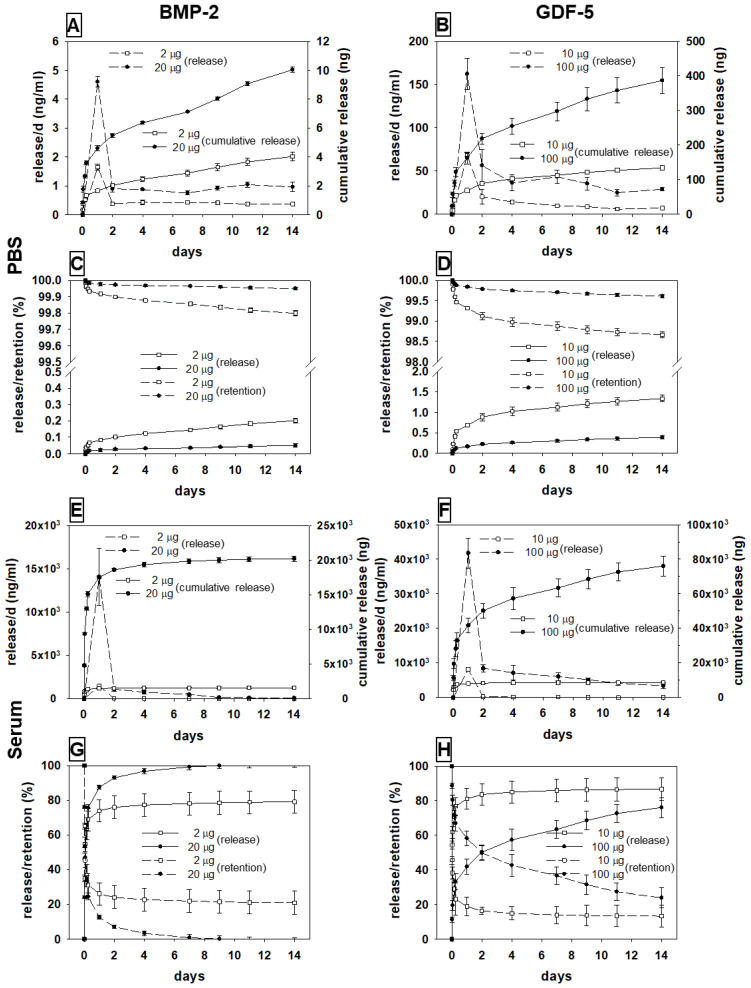
In vitro release per day (respective broken lines; left axis) and cumulative release (respective solid lines; right axis) of the two different doses of BMP-2 ((**A**); squares for 2 µg, circles for 20 µg) or GDF-5 ((**B**); squares for 10 µg, circles for 100 µg) from the therapeutically applied hydroxyapatite (HA) particles in PBS; release and retention (%) of BMP-2 (**C**) or GDF-5 (**D**) in PBS; in vitro release per day and cumulative release of BMP-2 (**E**) or GDF-5 (**F**) from the therapeutically applied HA particles in sheep serum; release and retention (%) of BMP-2 (**G**) or GDF-5 (**H**) in sheep serum.

The release of the two different growth factors from the HA particles in PBS or serum showed typical kinetics characterized by an initial burst/peak release until day 2, followed by a slower sustained release, and a minor release thereafter (for details, see Figure 3 and Table S1).

#### 3.2.1. BMP-2

##### Release in PBS

For low-dose BMP-2 (2 µg/5 mg HA), the cumulative release increased to 4 ng within 14 days (Figure 3A), resulting in a release of only 0.2% (Figure 3C; Table S1). For high-dose BMP-2 (20 µg), the cumulative release increased to 10 ng within 14 days (release of only 0.05% of the applied dose; Figure 3A,C).

##### Release in Sheep Serum

Incubation with sheep serum strongly augmented the release of both BMP-2 concentrations, with a stronger initial burst and a more gradual return to a low-release plateau than seen with PBS (Figure 3A,E; Table S1).

For low-dose BMP-2 (2 µg), the cumulative release rose to 1580 ng within 14 days (Figure 3E), corresponding to a release of approx. 80% (Figure 3G; Table S1). A similar time course was observed for the high-dose BMP-2 (20 µg), with an increase to 20,000 ng within 14 days (100%; Figure 3E,G).

#### 3.2.2. GDF-5

##### Release in PBS

For low-dose GDF-5 (10 µg/5 mg HA), the cumulative release amounted to 134 ng within 14 days (Figure 3B), corresponding to a release of only 1.3% (Figure 3D; Table S1). For high-dose GDF-5 (100 µg), the cumulative release was 387 ng in 14 days (0.4% of the applied dose; Figure 3B,D).

##### Release in Sheep Serum

For both dosages, the release was again strongly augmented by incubation in sheep serum, with a stronger initial burst and a slower return to a low-release plateau than observed with PBS (Figure 3B,F).

For low-dose GDF-5 (10 µg), the cumulative release amounted to 8670 ng within 14 days (Figure 3F), indicating a release of approx. 87% of the applied dose (Figure 3H; Table S1). This was also observed for high-dose GDF-5 (100 µg), with an increase to 76,000 ng within 14 days (76%; Figure 1H and Figure 3F; Table S1).

### 3.3. Distribution of Injected HA Particles; Potential Side Effects of BMP Therapy

**Figure 4:**
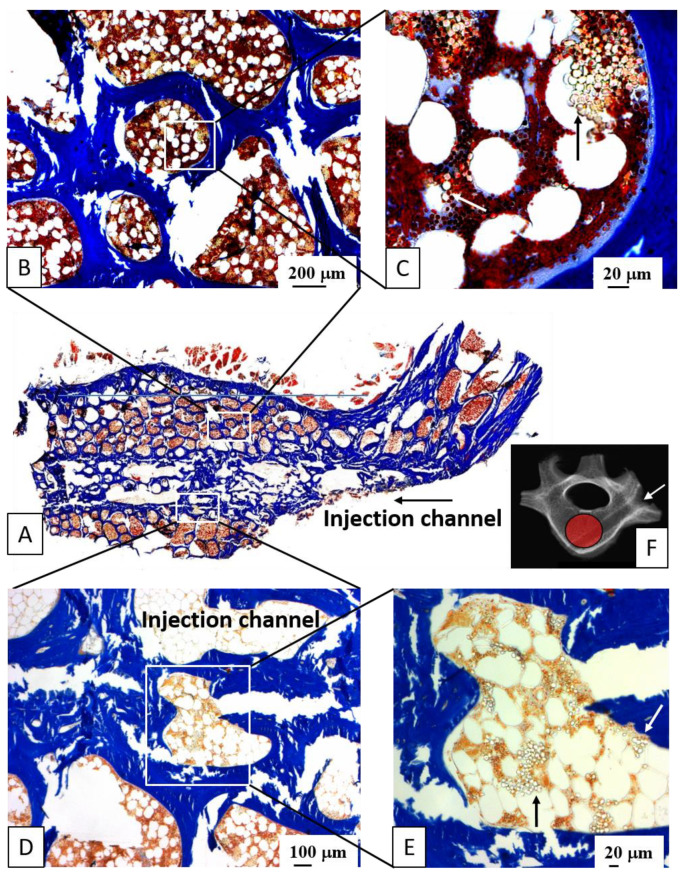
Light field (**A**,**B**,**D**) and phase contrast histology images (**C**,**E**) of therapeutically injected hydroxyapatite (HA) particles (see arrows in (**C**,**E**)) in plastic-embedded sections of lumbar vertebral bodies (L4 = HA/BMP-2; 10 μg; 3 months); trichrome stain according to Masson-Goldner, staining bone trabeculae including newly formed bone, as well as corticalis in blue, bone marrow including hematopoietic cells and fat marrow in brown/white, and HA particles in contrasted white (see arrows); (**F**) macro X-ray of the injected vertebral body demonstrating the placement of the injection channel (white arrow) and the spongiosa cylinder harvested for biomechanical analysis (red circle).

The injected carrier material (HA) was clearly detectable in plastic-embedded sections of all animals as rounded, non-staining particles. These were located in the immediate vicinity of the injection channel and within approx. 5 mm of its edge, as well as at the surface of the trabeculae and diffusely in the bone marrow (Figure 4).

In decalcified paraffin sections, there were no signs of inflammatory infiltration with polymorphonuclear neutrophilic leukocytes, lymphocytes, or macrophages in any of the groups or locations, indicating the absence of a foreign body reaction. Moreover, the vertebral bodies of all groups showed an equally active hematopoietic bone marrow concerning the lymphoid and myeloid lineage (thrombopoiesis; erythropoiesis), thus excluding any significant effect of the therapy on hematopoiesis. Finally, there were no signs of ectopic bone formation at the entry of the injection channel or outside the vertebral bodies in any group.

### 3.4. Single Injection of HA-Bound BMP-2 or GDF-5 Induces Long-Term Bone Formation—Global Analysis of the Therapeutic Effects of Carrier HA or BMP-Coated Carrier

To address the working hypothesis that global effects of treatment (group), time point (time), growth factor dose (dose), and distance from the injection channel (area) were present, a linear mixed effects model was used to analyze the influence of these main effects, as well as the interactions between group on one hand and the parameters time, dose, and area on the other hand, on the ten different static and dynamic histomorphometrical parameters (Table 2).

A highly significant influence of the main effect group (i.e., therapy with HA or HA/BMPs) with a large or medium effect size on most of the density and histological parameters (9/10 and 8/10 parameters for BMP-2 and GDF-5, respectively; Table 2 and Table S2) clearly indicated efficacious induction of bone formation by therapy with the carrier HA and/or HA coated with either BMP-2 or GDF-5. Post hoc analyses confirmed such therapeutic effects in area 1 (adjacent to the injection channel) for HA versus control in the BMP-2 and GDF-5 groups (6/10 and 4/10 parameters, respectively) and for HA/BMP-2 and HA/GDF-5 versus control (6/10 and 8/10 parameters, respectively; Table 2 and Table S2), with consistently larger effects for HA/BMPs than for HA. In the remote area 2, the therapeutic effects of HA and HA/BMPs were also supported, but less clearly and by fewer parameters.

A significant influence of the main effect time (i.e., long-term time point 6 or 9 months versus short-term time point 3 months after therapy) was also clearly supported in the case of GDF-5 (8/10 parameters with a large effect size), but less clearly for BMP-2 (3/10 parameters with a large effect size; Table 2 and Table S2).

A significant influence of the main effect dose (i.e., respective low and high dose of BMP-2 and GDF-5) was less likely, since it was only supported by a few, partially paradoxical effects for BMP-2 or GDF-5 (Table 2 and Table S2), indicating that low-dose therapy may be sufficient.

In contrast, the main effect area again showed a significant influence on most of the parameters in the BMP-2 and GDF-5 groups (4/5 and 5/5 parameters, respectively; Table 2 and Table S2), suggesting that the therapeutic effect decreases with increasing distance from the site of HA particle application.

Significant interactions with a high effect size between group*area for selected parameters in the BMP-2 group (BV/TV and Tb.Th) and the GDF-5 group (BV/TV, Tb.Th, and OS/BS) reflect the fact that significant differences between the adjacent area 1 and the remote area 2 were only present in the HA and/or HA/BMP groups, but not in the untouched control groups (Table 2 and Table S2). Interactions between group*time and group*dose were only significant for a few parameters, showed a small to medium effect size, and were partially paradoxical (Table S2).

**Figure 5:**
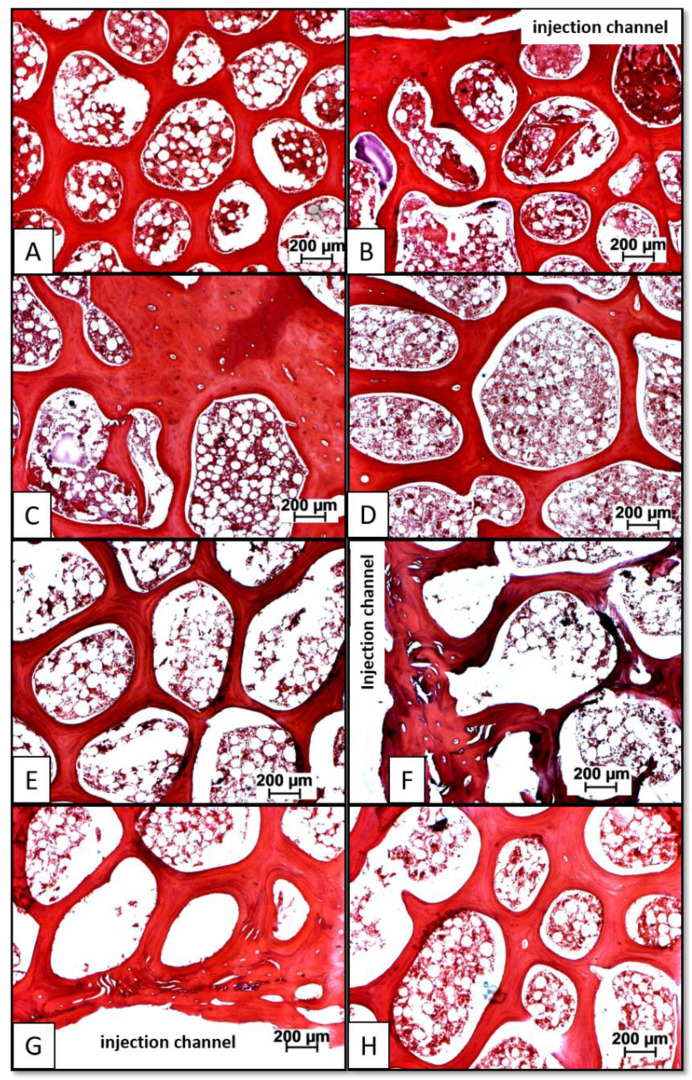
Paraffin sections of lumbar vertebral bodies from long-term animals (9 months) injected with either 10 µg BMP-2 (high dose; (**A**–**D**)) or with 5 µg GDF-5 (low dose; (**E**–**H**); for a low magnification overview of the injection channel in the vertebral body please compare with Figure 4). Hematoxylin-eosin stain, staining bone trabeculae including newly formed bone with embedded osteocytes in red and bone marrow including hematopoietic cells and fat marrow in purple/white; (**A**) control (L3); (**B**) HA-injected vertebral body (L5; injection channel); (**C**) HA/BMP-2-injected vertebral body (L4; injection channel); (**D**) HA/BMP-2-injected vertebral body (L4; remote area); (**E**) control (L3); (**F**) HA-injected vertebral body (L5; injection channel); (**G**) HA/GDF-5-injected vertebral body (L4; injection channel); (**H**) HA/GDF-5-injected vertebral body (L4; remote area).

**Figure 6:**
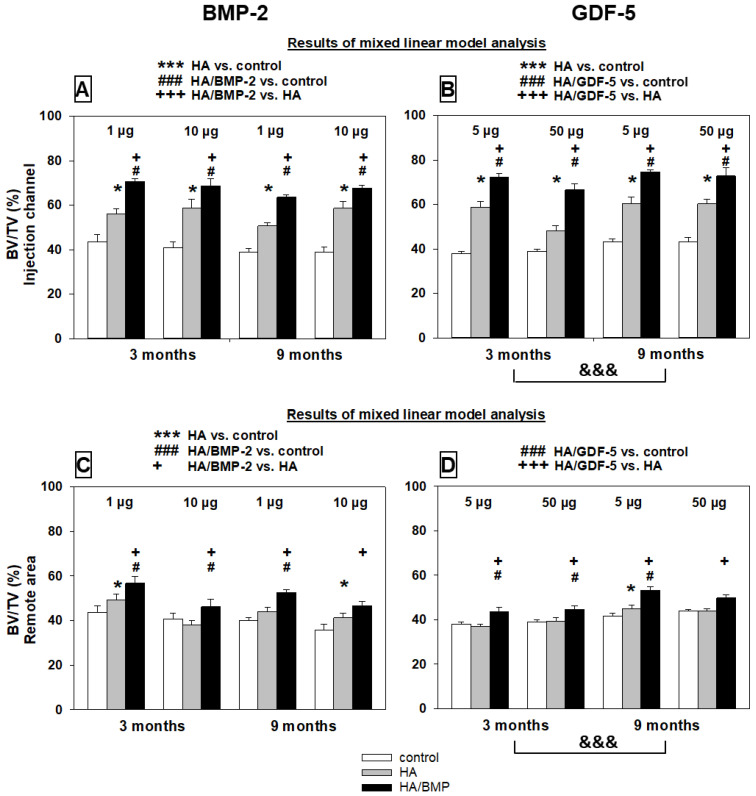
Increase of bone volume (%) in paraffin sections of lumbar vertebral bodies (L3 = control; L4 = HA/BMP; L5 = HA) of all investigated groups in the vicinity of the injection channel (**A**,**B**) and in the remote area (**C**,**D**). Results of the mixed linear model analysis (compare with Table 2 and Table S2); *** *p* < 0.001, * *p* ≤ 0.05 for HA compared to non-injected control; ### *p* < 0.001, # *p* ≤ 0.05 for HA/BMP compared to non-injected control; and +++ *p* < 0.001, + *p* ≤ 0.05 for HA/BMP compared to HA; &&& *p* < 0.001 for 9 months compared to 3 months. Please note: The linear mixed effects model was used to analyze global effects and overall interactions of the main effects treatment (group), time point (time), BMP dose (dose), and distance from the injection channel (area); subsequently, the non-parametric multigroup Kruskal–Wallis and two-sided Wilcoxon tests were applied for the comparison of paired samples within one group, and the multigroup Friedman and two-sided Mann-Whitney U-tests for statistical evaluation of differences between groups with the above-mentioned significance levels.

**Figure 7:**
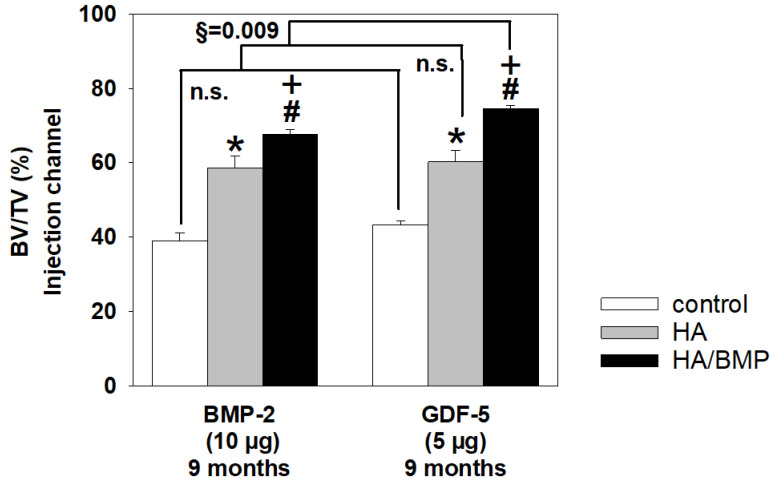
Increase of bone volume (%) in paraffin sections of lumbar vertebral bodies (L3 = control; L4 = HA/BMP; L5 = HA) of selected BMP-2 and GDF-5 groups in the vicinity of the injection channel. n.s. = not significant; *p* ≤ 0.05 Wilcoxon test, * for HA compared to non-injected control; # for HA/BMP compared to non-injected control; and + for HA/BMP compared to HA; brackets above the bars indicate the direct statistical comparison of the respective vertebral bodies in the BMP-2 and GDF-5 groups (§ *p* ≤ 0.01 U-test).

**Figure 8:**
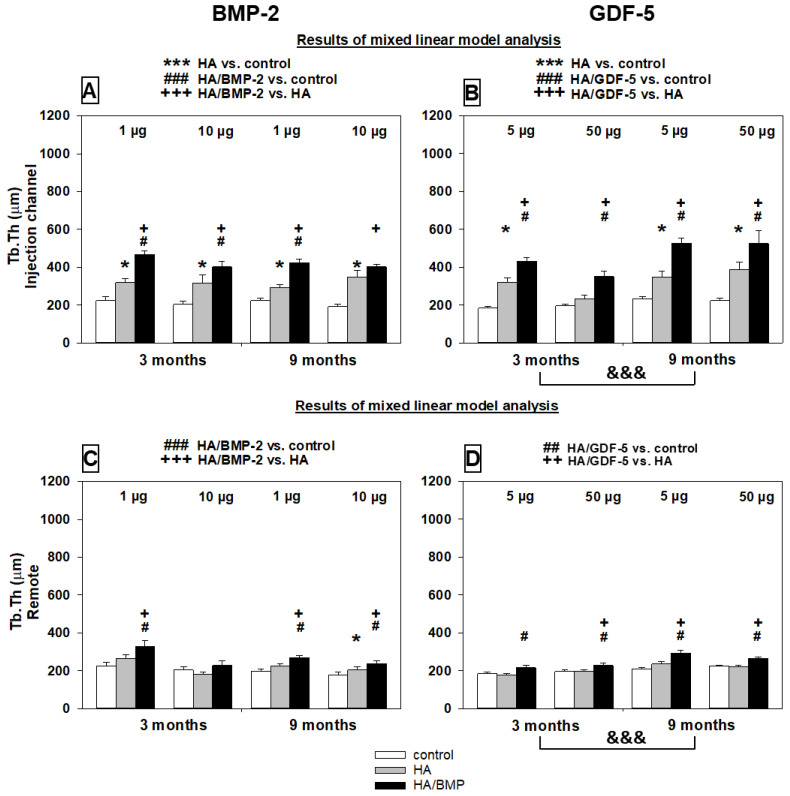
Increase of trabecular thickness (µm) in paraffin sections of lumbar vertebral bodies (L3 = control, L4 = HA/BMP; L5 = HA) of all investigated groups in the injection channel (**A**,**B**) and the remote area (**C**,**D**). Results of the mixed linear model analysis (compare with Table 2 and Table S2); *** *p* < 0.001, * *p* ≤ 0.05 for HA compared to non-injected control; ### *p* < 0.001, ## *p* ≤ 0.01, # *p* ≤ 0.05 for HA/BMP compared to non-injected control; and +++ *p* < 0.001, ++ *p* ≤ 0.01, + *p* ≤ 0.05 for HA/BMP compared to HA; &&& *p* < 0.001 for 9 months compared to 3 months. Please note: The linear mixed effects model was used to analyze global effects and overall interactions of the main effects treatment (group), time point (time), BMP dose (dose), and distance from the injection channel (area); subsequently, the non-parametric multigroup Kruskal-Wallis and two-sided Wilcoxon tests were applied for the comparison of paired samples within one group, and the multigroup Friedman and two-sided Mann-Whitney U-tests for statistical evaluation of differences between groups with the above-mentioned significance levels.

**Figure 9:**
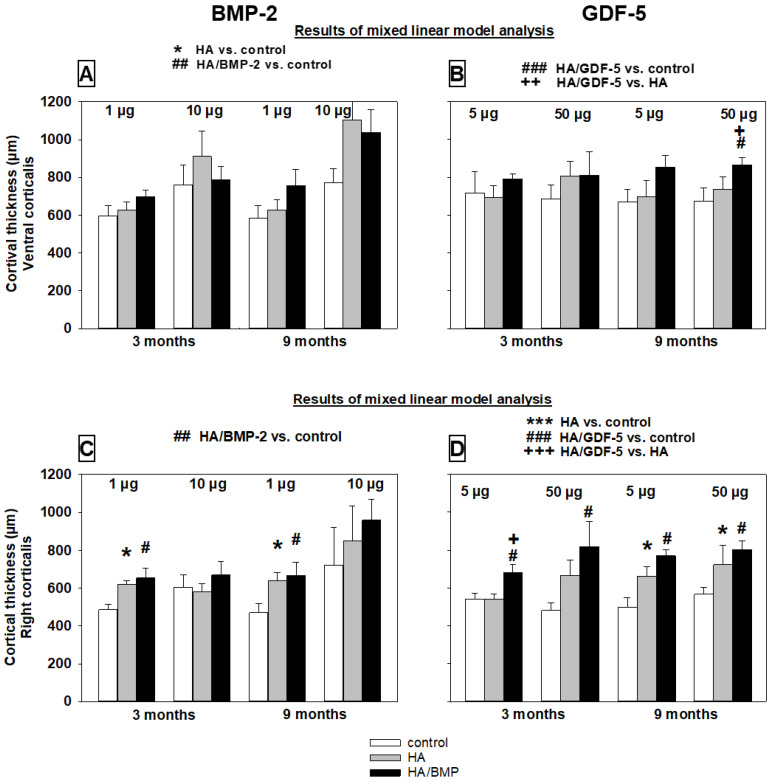
Increase of cortical thickness (µm) in paraffin sections of lumbar vertebral bodies (L3 = control, L4 = HA/BMP; L5 = HA) of all investigated groups in the ventral corticalis close to the tip of the injection channel (**A**,**B**) and the right corticalis (**C**,**D**). Results of the mixed linear model analysis (compare with Table 2 and Table S2); *** *p* < 0.001, * *p* ≤ 0.05 for HA compared to non-injected control; ### *p* < 0.001, ## *p* ≤ 0.01, # *p* ≤ 0.05 for HA/BMP compared to non-injected control; and +++ *p* < 0.001, ++ *p* ≤ 0.01, + *p* ≤ 0.05 for HA/BMP compared to HA. Please note: The linear mixed effects model was used to analyze global effects and overall interactions of the main effects treatment (group), time point (time), BMP dose (dose), and distance from the injection channel (area); subsequently, the non-parametric multigroup Kruskal-Wallis and two-sided Wilcoxon tests were applied for the comparison of paired samples within one group, and the multigroup Friedman and two-sided Mann-Whitney U-tests for statistical evaluation of differences between groups with the above-mentioned significance levels.

**Figure 10:**
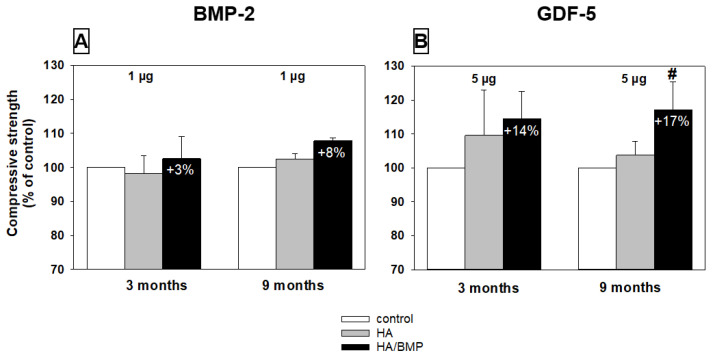
Relative increase of compressive strength (MPa; expressed as % of control) in lumbar vertebral bodies (L3 = control, L4 = HA/BMP; L5 = HA) of four selected groups (3 and 9 months; low-dose BMP-2 (**A**) or GDF-5 (**B**)); *p* ≤ 0.05 Wilcoxon test; # for HA/BMP compared to non-injected control; the HA/GDF-5 particles induced a numerical 14% or significant 17% increase at 3 and 9 months, respectively.

The results of global effects with the linear mixed effects model were largely confirmed by the explorative analysis with the non-parametric multigroup Kruskal-Wallis and two-sided Wilcoxon tests for the comparison of paired samples within one group, and the multigroup Friedman and two-sided Mann-Whitney U-tests for statistical evaluation of differences between groups (Figure 5, Figure 6, Figure 7, Figure 8, Figure 9, Figure 10 and Figure S2).

### 3.5. Bone Mineral Density

Based on the proven global treatment effects (Table 2), exploratory analyses were also performed to assess single group comparisons.

HA/BMP-injected lumbar vertebral bodies of all groups showed significantly increased bone mineral density in comparison to HA-injected and/or non-injected control vertebral bodies (Table 2 and Table S2; Figure S2A,B). These effects were observed for both BMP-2 and GDF-5. The significant differences were considered to be due to genuine therapeutic effects of the HA particles ±BMP (and not to the pure presence of HA particles), since the ex vivo injection of uncoated HA particles into the L3 vertebral body of 10 untreated cadaver sheep did not significantly change the osteodensitometry values.

### 3.6. Static Histology/Histomorphometry

#### 3.6.1. Structural Parameters

##### Bone Volume

For both BMP-2 and GDF-5, the bone volume values were significantly higher in HA/BMP-injected vertebrae than in either HA-injected or non-injected control vertebrae (see Figure 5A–H for paraffin sections; Table 2 and Table S2; Figure 6A–D). For HA/BMP-2, there was no clear influence of the main effects time and dose, showing that the significant influence of treatment with HA/BMP-2 is independent of the investigated time point and BMP dose. In the case of HA/GDF-5, in contrast, the main effect time showed a significant influence on the bone volume with higher values for the 9-month time point (Figure 6B,D), indicating long-term effects of a single time local treatment.

Treatment effects were observed in both investigated localizations (injection channel; remote area, center of the vertebrae, distance from the injection channel approx. 10 mm), indicating that the treatment effect is not confined to the immediate vicinity of the injection channel (Table 2 and Table S2; Figure 6A–D). However, the consistently significant influence of the main effect area for both BMPs with lower values in the remote area shows that there is a functional gradient of the treatment effects (Table 2 and Table S2; compare Figure 6A with C and Figure 6B with D).

Overall increase of the bone volume by HA/BMP treatment versus controls irrespective of the applied BMP, dose, and time point (*n* = 48 animals) was 71% for the injection channel (Figure 6A,B) and 22% for the remote area (Figure 6C,D).

Notably, the effects of 5 µg HA/GDF-5 on the bone volume at 9 months were significantly more pronounced than those of 10 µg HA/BMP-2 (Figure 7), although only half the molar dose of rhGFD-5 was applied (on the basis of an almost identical molecular weight of the two growth factors). This difference was specific for the HA/BMP treatment, since neither the untreated controls nor the HA groups showed such significant differences (Figure 7).

##### Trabecular Thickness

Similarly to the bone volume, the trabecular thickness was significantly higher in the groups treated with BMP-coated HA particles than with HA alone or non-injected controls (Table 2 and Table S2; see details in Figure 8A–D). Again, for HA/BMP-2 there was no significant influence of the main effects time and dose, indicating that the significant influence of treatment with HA/BMP-2 is valid for both time points and doses. For HA/GDF-5, a significant influence of the main effect time with higher values for the 9-month time point again suggested long-term effects of a single initial treatment (Figure 8B,D). For both BMP-2 and GDF-5, the significant influence of the main effect area with lower values for the remote area indicated a functional gradient of the treatment effects (Table 2 and Table S2; compare Figure 8A with C and Figure 8B with D).

Overall increase of the trabecular thickness by HA/BMP treatment versus controls irrespective of the applied BMP, dose, and time point (*n* = 48 animals) was 110% for the injection channel (Figure 8A,B) and 22% for the remote area (Figure 8C,D).

##### Cortical Thickness

The cortical thickness on the ventral side of the vertebrae (close to the tip of the injection channel) and the right side was mostly higher in the groups treated with BMP-coated HA particles than with HA alone or non-injected controls (Table 2 and Table S2; see details in Figure 9A–D). The lack of a clear and consistent influence of the main effects time and dose (compare with global analyses in Section 3.4) suggested that the significant influence of treatment with HA/BMPs is valid for both time points and doses. For both BMP-2 and GDF-5, the significant influence of the main effect area with lower values for the more remote area right corticalis indicated a functional gradient of the treatment effects (Table 2 and Table S2; compare Figure 9A with C; Figure 9B with D).

Overall increase of the cortical thickness by HA/BMP treatment versus controls irrespective of the applied BMP, dose, and time point (*n* = 48 animals) was 37% for the injection channel (Figure 8A,B) and 21% for the remote area (Figure 8C,D).

#### 3.6.2. Bone Formation Parameters (Static/Dynamic Histomorphometry)

For both BMP-2 and GDF-5, the bone formation parameters (i.e., osteoblast surface, osteoid surface, mineralized surface, mineral apposition rate, and bone formation rate) did not consistently differ between HA/BMP-injected samples and HA-injected or non-injected controls (Table 3). However, a highly significant influence of the main effect group with a large or medium effect size for most of the bone formation parameters (5/5 and 4/5 parameters for BMP-2 and GDF-5, respectively; Table 2 and Table S2) strongly suggested the therapeutic induction of bone formation by the carrier HA and/or either BMP-2 or GDF-5.

A significant influence of the main effect time was also clearly supported for GDF-5 (5/5 parameters) and, less clearly, for BMP-2 (2/5 parameters; Table 2 and Table S2).

A significant influence of the main effect dose was again unlikely and only supported by a few, partially paradoxical effects for BMP-2 or GDF-5 (Table 2 and Table S2).

The main effect area instead showed a significant influence in the BMP-2 and GDF-5 groups (1/2 and 2/2 parameters, respectively; Table 2 and Table S2), suggesting a decreasing therapeutic effect with increasing distance from the injection channel.

A significant interaction between group*area for the parameter OS/BS in the GDF-5 group reflects the finding that significant differences for this parameter between the adjacent area 1 and the remote area 2 were only present in the HA and/or HA/BMP groups, but not in the untouched control group (Table 2 and Table S2).

Interestingly, in several groups the injection of HA particles in L5 led to a reduction of the parameters mineralized surface, mineral apposition rate, and/or bone formation rate compared to non-injected control vertebrae (Table 3). This is possibly due to a negative competition between endogenous bone HA and the injected HA particles for the binding of the injected fluorescent marker OTC, in line with previous reports showing competitive binding of therapeutically applied tetracycline and bone calcium to bone collagen [69].

### 3.7. Biomechanical Testing (Compressive Strength)

Four selected groups were analyzed concerning the compressive strength of the vertebral spongiosa cylinders (short term and long term; low-dose BMP-2 or GDF-5; harvested cylinder illustrated in Figure 4F above). Notably, HA/GDF-5-injected vertebrae showed higher compressive strength in the short-term (on average +14%) and long-term groups (on average +17%; *p* ≤ 0.05) compared to the respective non-injected control vertebrae. For HA/BMP-2-injected vertebral bodies, the increases were more limited (+3% and +8%, respectively; Figure 10A,B).

## 4. Discussion

### 4.1. Animal Model

In the present study, aged female sheep (mean age 9 years) were used as an animal model for human senile osteopenia [34,40]. Compared to young ewes (2–4 years), the lumbar vertebral bodies of the animals show significantly decreased histological parameters of bone structure and bone formation, and increased bone resorption, resulting in numerically decreased biomechanical properties [40]. This sheep model represents an innovative approach, since most studies have thus far used young ovariectomized or hormonally pretreated ewes [53,70,71]. In the present study, in contrast, the ewes showed functional cycling ovaries as reported previously [61]; therefore, a post-menopausal influence on bone can be excluded.

### 4.2. In Vitro Growth Factor Release from the HA Particles

For both BMPs, the release in the frequently used experimental release medium PBS was very limited (maximally 1.3% within 14 days). This release was considerably lower than that in previous studies on the BMP-2 release from Ca-P carriers (e.g., [16,72]), likely due to different experimental conditions concerning the specific type of carrier, the quantity of loaded protein, and the release temperature (room temperature in the present study). However, such low release of comparable amounts of loaded BMP-2 (18.9 µg) was also observed at 37 °C in an independent experimental series, underlining the validity of the current data. For in vitro release studies, however, a study design including both PBS and protein-containing PBS, serum, or cell culture medium should be pursued to avoid an underestimation of the BMP release ([49]; see below).

On the other hand, the release of both BMPs was strongly augmented in sheep serum, with an increased initial peak release until day 2 and a more gradual return to a low-release plateau (up to 100% release within 14 days, also indicating complete releasability of the coated protein). This three-phase release pattern of initial burst, subsequent sustained release, and negligible release is similar to the pattern observed for other ceramic and calcium phosphate scaffolds [49,72,73,74] or collagen sponges [16]. In addition, the enhanced release in protein-rich serum concords with the increased release of BMP-2 from calcium phosphate or collagen-based scaffolds in protein-containing serum or protein-enriched PBS ([16] and references therein). This effect is considered to be due to interference with high affinity binding sites for BMP-2 (upon BMP-2 detachment and rebinding at chemical equilibrium) or with reciprocal electrostatic and hydrophobic interactions between BMPs and carriers ([16] and references therein). Candidate molecules mediating the augmented release in serum versus PBS include serum albumin, but also erythroferrone, a serum glycoprotein secreted by erythroblasts in response to stimulation by erythropoietin and possibly acting as a natural ligand trap for BMPs ([16] and references therein).

The BMP release pattern from HA particles shown in this study is thus comparable to that from clinically used collagen carriers, despite large experimental variability regarding carrier materials, protein loading, and release conditions [16,33,75,76,77,78]. Notably, the currently approved high-dose BMP formulations (3.5 to 12 mg) show an initial burst release held responsible for inflammatory and osteoclastic side effects; therefore, lower doses of applied BMPs (present study) and/or a tuning of this initial release by modifications of the carrier material are highly desirable [16,17,18,19].

Interestingly, the BMP release from the current HA particles was in principle also comparable to that from a biodegradable, brushite-forming calcium-phosphate-cement (CPC), with three phases of initial burst until day 2, subsequent sustained release, and marginal long-term release ([49] and references therein). However, there were also some differences between HA particles and the CPC, i.e., the initial burst release from the CPC was more sustained, sometimes even with a second peak between day 6 and day 9. In addition, the cumulative BMP release from the HA particles in serum rapidly reached 76–100% within 3–4 days, whereas the BMP release from the CPC over 14 days did not exceed 26% [49]. In analogy, some studies described an early bulk release from a “loose” compartment, followed by slower release from a more tightly packed second compartment only when the BMP-2 was surface adsorbed onto a preset CPC, but not when incorporated into the CPC [74]. However, when BMP-2 was mixed with other paste-like materials such as demineralized bone putty (DBM), similar release patterns were reported [73]. As a limitation, it has to be kept in mind that in vitro experiments likely do not reflect all cellular, enzymatic, and physicochemical factors acting in situ in the target tissue.

### 4.3. Induction of Bone Formation

Single application of HA particles coated with either BMP-2 or GDF-5 strongly and significantly induced bone formation, as demonstrated by histomorphometry, osteodensitometry, and biomechanics. The HA carrier material also induced significant bone formation, in agreement with the known osteoconductive effects of different calcium phosphate carriers ([11,12] and references therein); however, the effect of the growth-factor-coated HA was significantly stronger than that of the carrier alone for numerous parameters. These results clearly show that growth-factor-coated HA may be applicable to treat lumbar osteopenia, as previously reported for different growth-factor-coated bone grafting matrices in long bone fractures [62], spinal fusion [8,9,79], ulnar osteotomy [80], and necrosis of the femoral head [7]. Although the strongest effects of pure HA particles were noted in the immediate vicinity of the injection channel, the influence also extended to the remote area, likely due to mechanisms such as spreading of the HA particles (see below), ionic transfer to the surrounding protein-rich biological milieu, and possibly even osteoinductive properties of the calcium phosphate biomaterial [81,82,83].

Concerning the mode of ossification, HA or BMP-coated HA only induced signs of early enchondral ossification (i.e., islets of hyaline cartilage; see representative Figure S3), but not of intramembraneous ossification, although the presence of such ossification in other areas of the implantation bed cannot be completely excluded. For BMP-2, these results are in good agreement with some previous reports [84], but they are in contrast to a predominantly intramembraneous ossification observed for example around HA/BMP-2-coated titanium cylinders implanted into bone and fat marrow of the tibial head [34]. Among other factors, the particular cellular environment and micromilieu at the implantation site (including mesenchymal precursor recruitment) may provide an explanation for the different modes of ossification.

Concerning the osteopenic status of the aged ewes (compressive strength 81% of the value in young healthy sheep; [40]), low-dose HA/GDF-5 at 3 months induced significant biomechanical augmentation (from 21.4 MPa to 24.3 MPa, i.e., approx. 92% of the value in young healthy sheep). This effect was even stronger at 9 months (30.9 MPa, i.e., 117% compared to young controls). These results indicate that sustained augmentation of biomechanical properties from the level of aged osteopenic sheep to that of young control sheep may be achievable.

### 4.4. Effects of Very Low Doses of Growth Factors

An important result of our study was that low-dose treatment with BMP-2 or GDF-5 was already sufficient to induce bone formation in the lumbar spine and that the additional effects of higher doses were negligible. While the mechanisms of this phenomenon remain to be elucidated, lowering the threshold levels for successful augmentation of bone formation in animal models by BMP-2 or GDF-5 by a factor of 10-fold to 30-fold ([11,12,30] and references therein) may contribute to increased treatment safety.

Notably, the doses of growth factors used in the present study are several magnitudes lower (20,000-fold to 1000-fold) than those used in previous or current clinical BMP products for bone grafting surgery ([11,12] and references therein). If transferable to human osteoporosis or other bone disorders, the application of such low doses would have the major advantage of reducing possible side effects (e.g., ectopic bone formation, potential risk of osteosarcoma and autoantibody formation, or spinal complications [13,14,15]). In addition, cost-effectiveness of the resulting bone replacement product may also be improved.

### 4.5. Long-Term Efficacy of Growth-Factor-Coated HA

Despite a single intravertebral injection, several histological effects were still evident 9 months after treatment (in particular after injection of HA/GDF-5), indicating long-term efficacy of the growth factors on bone growth. Many parameters even further increased from the 3-month to the 9-month time point, in analogy to other bone grafting or defect surgery models [8,9,11,12,22,30,79,85,86,87]. To our knowledge, however, this is the first report of long-term effects of very low BMP doses in osteopenic vertebral bodies. This long-term efficacy may be at least partially due to the fact that recombinant BMP-2/GDF-5 was produced in a non-glycosylated form in *E. coli*, which shows lower solubility in physiological fluids ([88]) and may therefore remain at the site of application for a prolonged period of time. On the other hand, the non-glycosylated BMP-2/GDF-5 was rapidly released in vitro from the HA particles in serum over 14 days (release between 76% and 100%, depending on the BMP and its dose). If these results are a reasonably good estimate for the in vivo situation, an initially substantial, local release of low-dose BMPs may be a sufficient early trigger resulting in long-term stimulation of osteogenesis.

### 4.6. Remote Effects of the Therapy with Growth-Factor-Coated HA

As expected, the induction of bone formation close to the injection channel was generally higher than in the remote area (i.e., the center of the vertebral body). Nonetheless, the remote area of the vertebral bodies injected with growth-factor-coated HA still showed significantly stronger effects than non-injected or carrier-injected controls, possibly also supported by the spreading of the injected, BMP-coated HA particles within a distance of approx. 5 mm from the edge of the injection channel (see Figure 4). Therefore, despite their limited in vivo stability, both BMP-2 and GDF-5 appear to act over a considerable distance (approx. 10 mm) and the locally released growth factors may be effective in the spongiosa of the entire vertebral body. This result is of potential clinical relevance, for example in the case of bipedicular vertebroplasty/kyphoplasty for the treatment of osteoporotic fractures.

### 4.7. Direct Comparison of BMP-2 and GFD-5

Although the local release of the two different BMPs may not be comparable, the effects of 5 µg HA/GDF-5 in histomorphometry (e.g., bone volume) were more pronounced than those of 10 µg HA/BMP-2, although only half the molar dose of rhGFD-5 was applied (on the basis of an almost identical molecular weight of the two growth factors).

This difference was observed in some short-term and long-term groups, underlining the strong and long-lasting potential of GDF-5 (also called BMP-14) to induce bone formation. The present results are in contrast with some previous studies describing BMP-2 as the gold standard for its osteoinductive properties in bone grafting/defect surgery [26,39,87,89]. Our results, however, indicate that GDF-5 or its mutant BB-1 may be at least as effective for this purpose and may represent a valid option for bone grafting surgery [7,8,9,12,22,30,31].

### 4.8. Limitations of the Study

The present study lacked data on empty control defects without injected HA particles ± BMPs. However, empty vertebral defects of a similar size were analyzed in previous studies in the same model of aged osteopenic sheep [40], showing that structural parameters such as bone volume/total volume around the empty defect channel were consistently enhanced compared to untouched controls, but in general were significantly lower than for calcium phosphate cements without or with BMPs [64]. Thus, the current vertebral bone defect has a sufficient critical size to act as a control, and the significant augmentation of bone parameters truly reflects osteoinductive effects of the BMPs.

In the present study, the L3 vertebral body was systematically used as untouched control and the vertebral bodies L4 and L5 for the injection of HA particles with or without BMPs. While relevant differences in structural and mechanical parameters among the vertebral bodies L3–L5 should be considered, our own previous reports have failed to show statistically significant differences of structural bone parameters between L3, L4, and L5 in young sheep (2–4 years; [64]). Hence, a systematic effect of differential bone regenerative capability among the vertebral bodies L3 to L5 is rather unlikely.

In the present study, osteodensitometry showed a more limited sensitivity than histomorphometry to detect the influence of HA particles with or without BMPs on bone regeneration, in line with a low sensitivity of clinical BMD measurements for fracture prediction [90]. A possible explanation is that BMD measurements rely on a summation image of the bone density in the vertebral spongiosa along the longitudinal axis of the vertebral body, whereas histological sections allow detailed local analyses of bone structure and formation parameters in the bone tissue immediately adjacent to the injection channel. Similarly, standard anterior-posterior X-rays of the lumbar spine obtained every 3 months after surgery were not sensitive enough to reveal changes in the spongiosa density of the treated lumbar vertebrae, possible due to the high density of the surrounding corticalis. This limited sensitivity was only overcome when the covering and base plates were removed for osteodensitometry of the central, spongious area of the vertebral bodies in the longitudinal direction (see above).

The present approach to use plastic-embedded sections generated by sawing, grinding, and staining with Masson-Goldner trichrome to determine histological parameters of bone structure and formation limits the experimental possibilities, since only one section is obtained per vertebral body. Unfortunately, alternative equipment to directly cut thin sections of plastic-embedded undecalcified bones was not available in the laboratory at the time of the studies. However, the high reproducibility of the results of bone structure/regeneration in all groups indicates that this limitation did not substantially affect the validity of the present results.

## 5. Conclusions

The present study used a novel animal model of aged osteopenic sheep, independent of post-menopausal changes, and hence more generally reflecting senile osteopenia [40]. Osteopenia was efficiently treated by intravertebral injection of growth-factor-coated HA, with histological and biomechanical features approaching or even exceeding those observed in healthy young sheep. Long-lasting therapeutic effects were achieved with very low doses of recombinant growth factors, a promising result in view of minimizing possible side effects [15]. Moreover, the presence of clear therapeutic effects in areas remote from the application site may prove useful for surgical procedures such as bipedicular vertebroplasty/kyphoplasty, the therapy of pseudarthrosis with augmentation, the application of vertebral cages, and complicated or re-spondylodesis. Finally, due to its non-inferiority or even superiority to the gold standard BMP-2, the growth factor GDF-5 may represent a valid option for bone grafting surgery.

### Potential Clinical Relevance

Significant bone induction was observed with BMP-2 or GDF-5 doses substantially lower than previously applied in clinical practice (20,000-fold to 1000-fold lower). Although the present study was not designed as a safety study and therefore did not allow a systematic assessment of the safety profile of the BMP-coated HA particles, there were no signs of adverse effects, local inflammatory reactions, or alterations of adjacent hematopoiesis. Thus, the present low-dose approach may help in minimizing safety risks [15].

## Figures and Tables

**Table 1:** Time schedule of the animal experiments.

Growth Factor	Duration	Dose	BMP-2/GDF-5 Dosage
BMP-2	Short term * (3 months)	low	1 µg BMP-2/2.5 mg HA/50 µL serum (*n* = 12)
		high	10 µg BMP-2/2.5 mg HA/50 µL serum (*n* = 6)
	Long term * (9 months)	low	1 µg BMP-2/2.5 mg HA/50 µL serum (*n* = 12)
		high	10 µg BMP-2/2.5 mg HA/50 µL serum (*n* = 6)
GDF-5	Short term * (3 months)	low	5 µg GDF-5/2.5 mg HA/50 µL serum (*n* = 12)
		high	50 µg GDF-5/2.5 mg HA/50 µL serum (*n* = 6)
	Long term * (9 months)	low	5 µg GDF-5/2.5 mg HA/50 µL serum (*n* = 12)
		high	50 µg GDF-5/2.5 mg HA/50 µL serum (*n* = 6)

HA: hydroxyapatite particles; * 6 animals were only used for biomechanical testing.

**Table 2:** Results of the linear mixed effects model for treatment (group), time point (time), BMP dose (dose), and area-related effects of BMP-2 or GDF-5.

**Parameter**		**Treatment (Adjacent Area 1)**			**Treatment (Remote Area 2)**			**Group**	**Time**		**Dose**	**Area**	Group * Area
		**HA vs. Control**	**HA-BMP-2 vs. Control**	**HA-BMP-2 vs. HA**	**HA vs. Control**	**HA-BMP-2 vs. Control**	**HA-BMP-2 vs. HA**						
**BMD [%]** **(area 1)**	P (η_p_^2^)	n.s.	**0.001**	n.s.	n.a.	n.a.	n.a.	**0.001** (0.285)	n.s.		n.s.	n.a.	n.a.
**BV/TV [%]**	P (η_p_^2^)	**<0.001**	**<0.001**	**<0.001**	**<0.001**	**<0.001**	**0.032**	**<0.001** (0.749)	n.s.		n.s.	**<0.001** (0.551)	**<0.001** (0.375)
**Tb.Th [µm]**	P (η_p_^2^)	**<0.001**	**<0.001**	**<0.001**	n.s.	**<0.001**	**<0.001**	**<0.001** (0.730)	n.s.		n.s.	**<0.001** (0.629)	**<0.001** (0.442)
**Cort. Th [µm]**	P (η_p_^2^)	n.s.	**0.002**	n.s.	**0.014**	**0.007**	n.s.	**<0.001** (0.160)	n.s.		**0.011** (0.291)	**0.001** (0.114)	n.s.
**Ob.S/BS** **[%]**	P (η_p_^2^)	**0.004**	**<0.001**	n.s.	n.s.	n.s.	n.s.	**<0.001** (0.164)	**0.006** (0.340)		**0.047** (0.192)	n.s.	n.s.
**OS/BS** **[%]**	P (η_p_^2^)	**0.014**	**0.001**	n.s.	n.s.	n.s.	n.s.	**0.002** (0.137)	n.s.	n.s.		**0.045** (0.046)	n.s.
**MS/BS [X]** **(area 1)**	P (η_p_^2^)	n.s.	n.s.	**0.017**	n.a.	n.a.	n.a.	**0.015** (0.260)	n.s.		n.s.	n.a.	n.a.
**MAR [X]** **(area 1)**	P (η_p_^2^)	**0.043**	n.s.	**0.014**	n.a.	n.a.	n.a.	**0.015** (0.259)	**0.038** (0.273)		n.s.	n.a.	n.a.
**BFR/BS [X]** **(area 1)**	P (η_p_^2^)	**0.030**	n.s.	**0.019**	n.a.	n.a.	n.a.	**0.012** (0.270)	n.s.		n.s.	n.a.	n.a.
**ES/BS** **[%]**	P (η_p_^2^)	n.s.	n.s.	n.s.	n.s.	n.s.	n.s.	n.s.	**0.030** (0.262)		n.s.	n.s.	n.s.
**Parameter**		**Treatment (Adjacent Area 1)**			**Treatment (Remote Area 2)**			**Group**	**Time**		**Dose**	**Area**	Group * Area
		**HA vs. Control**	**HA-GDF-5 vs. Control**	**HA-GDF-5 vs. HA**	**HA vs. Control**	**HA-GDF-5 vs. Control**	**HA-GDF-5 vs. HA**						
**BMD [%]** **(area 1)**	P (η_p_^2^)	n.s.	**<0.001**	**0.003**	n.a.	n.a.	n.a.	**<0.001** (0.332)	n.s.		n.s.	n.a.	n.a.
**BV/TV [%]**	P (η_p_^2^)	**<0.001**	**<0.001**	**<0.001**	n.s.	**<0.001**	**<0.001**	**<0.001** (0.805)	**<0.001** (0.662)		n.s.	**<0.001** (0.551)	**<0.001** (0.375)
**Tb.Th [µm]**	P (η_p_^2^)	**<0.001**	**<0.001**	**<0.001**	n.s.	**0.003**	**0.007**	**<0.001** (0.664)	**<0.001** (0.633)		n.s.	**<0.001** (0.625)	**<0.001** (0.486)
**Cort. Th [µm]**	P (η_p_^2^)	**0.001**	**<0.001**	**<0.001**	n.s.	**<0.001**	**0.007**	**<0.001** (0.363)	n.s.		n.s.	**<0.001** (0.199)	n.s.
**Ob.S/BS** **[%]**	P (η_p_^2^)	n.s.	**0.003**	n.s.	n.s.	n.s.	n.s.	**0.006** (0.097)	**0.001** (0.420)		n.s.	**<0.001** (0.189)	n.s.
**OS/BS** **[%]**	P (η_p_^2^)	**<0.001**	**<0.001**	n.s.	n.s.	n.s.	n.s.	**<0.001** (0.274)	**<0.001** (0.763)		n.s.	**<0.001** (0.188)	**0.003** (0.107)
**MS/BS [X]** **(area 1)**	P (η_p_^2^)	n.s.	n.s.	n.s.	n.s.	n.s.	n.s.	n.s.	**<0.001** (0.679)		n.s.	n.a.	n.a.
**MAR [X]** **(area 1)**	P (η_p_^2^)	n.s.	**<0.001**	**<0.001**	n.a.	n.a.	n.a.	**<0.001** (0.310)	**0.001** (0.194)		**0.047** (0.071)	n.a.	n.a.
**BFR/BS [X]** **(area 1)**	P (η_p_^2^)	n.s.	**0.003**	**0.002**	n.a.	n.a.	n.a.	**0.002** (0.298)	**<0.001** (0.602)		n.s.	n.a.	n.a.
**ES/BS** **[%]**	P (η_p_^2^)	n.s.	n.s.	n.s.	n.s.	n.s.	n.s.	n.s.	**0.001** (0.472)		n.s.	n.s.	n.s.

Bone mineral density (BMD); bone volume/total volume (BV/TV); trabecular thickness (Tb.Th); osteoblast surface (Ob.S/BS); osteoid surface (OS/BS); cortical thickness (Cort. Th); mineralizing surface per bone surface (MS/BS); mineral apposition rate (MAR); bone formation rate surface-based BFR/BS; and eroded surface (ES/BS); η_p_^2^ = partial eta squared; HA = hydroxyapatite particles; BMP-2 = bone morphogenetic protein-2; GDF-5 = growth/differentiation factor-5; n.s. = not significant; n.a. = not applicable. A linear mixed effects model was fitted to compare the effects of treatment (group), time point after treatment (time), BMP dose (dose), and distance from the injection channel (area), as well as the interactions (*) between group on one hand and the parameters time, dose, and area on the other hand, on the ten different static and dynamic histomorphometrical parameters. Interactions between group * time and group * dose were only significant for a few parameters, showed a small to medium effect size, and were partially paradoxical (compare with Table S2). In this analysis, group, time, dose, and area were modeled as fixed effects with a random intercept per sheep, in order to account for multiple measurements per sheep and the correlation of these observations. η_p_^2^ from the model was used to assess the effect size (classification: 0.01 ≤ η_p_^2^ < 0.06 small effect size; 0.06 ≤ η_p_^2^ < 0.14 medium effect size; η_p_^2^ ≥ 0.14 large effect size). Indicated in bold: Values of *p* ≤ 0.05 for the influence of the fixed effects group, time, dose, and area or the interactions (*) between group and the parameters time, dose, and area.

**Table 3:** Control, HA, and HA/BMP vertebral bodies grouped by BMP, dose, and duration.

Group		Parameter	Injection Channel			Remote Area		
			Control (C)	HA	HA/BMP	Control (C)	HA	HA/BMP
**BMP-2** 3 months	1 µg	Ob.S/BS [%]	0.91 ± 0.35	1.94 ± 0.65 *	**2.00 ± 0.37 #**	1.02 ± 0.37	1.14 ± 0.16	1.21 ± 0.09
		OS/BS [%]	8.44 ± 6.80	12.79 ± 4.18	14.95 ± 6.71	8.44 ± 6.80	14.87 ± 6.86	23.32 ± 13.39
		MS/BS [x-fold]	1.00 ± 0.00	0.64 ± 0.20	0.91 ± 0.26	n. a.	n. a.	n. a.
		MAR [x-fold]	1.00 ± 0.00	0.55 ± 0.23	**1.18 ± 0.19 +**	n. a.	n. a.	n. a.
		BFR/BS [x-fold]	1.00 ± 0.00	0.50 ± 0.25 *	**1.09 ± 0.27 +**	n. a.	n. a.	n. a.
	10 µg	Ob.S/BS [%]	0.87 ± 0.29	1.19 ± 0.22	1.05 ± 0.12	0.87 ± 0.29	1.40 ± 0.31	1.40 ± 0.30
		OS/BS [%]	1.94 ± 1.20	9.99 ± 3.60	**19.45 ± 7.07 #**	1.94 ± 1.20	0.23 ± 0.21	3.10 ± 2.78
		MS/BS [x-fold]	1.00 ± 0.00	0.72 ± 0.31	1.22 ± 0.34	n. a.	n. a.	n. a.
		MAR [x-fold]	1.00 ± 0.00	0.37 ± 0.21	1.10 ± 0.20	n. a.	n. a.	n. a.
		BFR/BS [x-fold]	1.00 ± 0.00	0.37 ± 0.31	1.28 ± 0.28	n. a.	n. a.	n. a.
**GDF-5** 3 months	5 µg	Ob.S/BS [%]	0.90 ± 0.10	1.53 ± 0.27 *	**1.42 ± 0.20 #**	0.90 ± 0.10	0.84 ± 0.21	**1.22 ± 0.19 +**
		OS/BS [%]	10.45 ± 4.36	21.62 ± 3.02	**34.28 ± 4.59 #+**	10.45 ± 3.98	17.04 ± 4.45	14.46 ± 3.68
		MS/BS [x-fold]	1.00 ± 0.00	1.01 ± 0.57	1.16 ± 0.08	n. a.	n. a.	n. a.
		MAR [x-fold]	1.00 ± 0.00	0.00 ± 0.00 *	0.65 ± 0.34	n. a.	n. a.	n. a.
		BFR/BS [x-fold]	1.00 ± 0.00	0.00 ± 0.00 *	0.44 ± 0.26	n. a.	n. a.	n. a.
	50 µg	Ob.S/BS [%]	1.18 ± 0.09	1.41 ± 0.18	1.31 ± 0.26	1.18 ± 0.09	1.30 ± 0.26	1.39 ± 0.09
		OS/BS [%]	2.78 ± 1.80	30.60 ± 7.31 *	**26.36 ± 9.44 #**	2.78 ± 1.80	6.06 ± 1.84	**13.96 ± 4.41 #**
		MS/BS [x-fold]	1.00 ± 0.00	1.76 ± 0.27 *	2.24 ± 0.54	n. a.	n. a.	n. a.
		MAR [x-fold]	1.00 ± 0.00	8.00 ± 8.00	8.00 ± 8.00	n. a.	n. a.	n. a.
		BFR/BS [x-fold]	1.00 ± 0.00	8.00 ± 8.00	8.00 ± 8.00 #	n. a.	n. a.	n. a.
**BMP-2**	1 µg 9 mths.	Ob.S/BS [%]	1.77 ± 0.49	3.10 ± 0.47	**4.01 ± 1.10 #+**	2.86 ± 0.54	2.94 ± 0.48	**3.84 ± 0.48 &**
		OS/BS [%]	0.00 ± 0.00	19.18 ± 8.96	21.18 ± 8.85	0.00 ± 0.00	1.92 ± 1.11	1.79 ± 1.79
	1 µg 6 mths.	MS/BS [x-fold]	1.00 ± 0.00	0.88 ± 0.28	0.93 ± 0.27	n. a.	n. a.	n. a.
		MAR [x-fold]	1.00 ± 0.00	0.76 ± 0.26	0.95 ± 0.34	n. a.	n. a.	n. a.
		BFR/BS [x-fold]	1.00 ± 0.00	0.65 ± 0.23	0.73 ± 0.39	n. a.	n. a.	n. a.
	10 µg 9 mths.	Ob.S/BS [%]	1.41 ± 0.25	1.53 ± 0.29	2.11 ± 0.61	1.62 ± 0.34	1.66 ± 0.22	1.85 ± 0.17
		OS/BS [%]	0.00 ± 0.00	3.20 ± 3.20	3.94 ± 2.48	0.00 ± 0.00	0.12 ± 0.12	**1.80 ± 0.52 #**
	10 µg 6 mths.	MS/BS [x-fold]	1.00 ± 0.00	0.91 ± 0.15	1.35 ± 0.24	n. a.	n. a.	n. a.
		MAR [x-fold]	1.00 ± 0.00	0.59 ± 0.04 *	**1.14 ± 0.14 +**	n. a.	n. a.	n. a.
		BFR/BS [x-fold]	1.00 ± 0.00	0.56 ± 0.11 *	**1.61 ± 0.46 +**	n. a.	n. a.	n. a.
GDF-5	5 µg 9 mths.	Ob.S/BS [%]	3.00 ± 0.70	2.99 ± 0.76	**4.53 ± 1.32 &**	1.46 ± 0.15	1.53 ± 0.25	2.06 ± 0.40
		OS/BS [%]	0.38 ± 0.38	8.03 ± 3.09 *	**7.93 ± 3.11 #**	0.38 ± 0.38	0.50 ± 0.50	1.08 ± 0.72
	5 µg 6 mths.	MS/BS [x-fold]	1.00 ± 0.00	0.87 ± 0.15	1.03 ± 0.10	n. a.	n. a.	n. a.
		MAR [x-fold]	1.00 ± 0.00	**1.08 ± 0.22 &&**	**2.03 ± 0.13 #+&&**	n. a.	n. a.	n. a.
		BFR/BS [x-fold]	1.00 ± 0.00	**0.80 ± 0.21 &&**	**1.74 ± 0.20 +&&**	n. a.	n. a.	n. a.
	50 µg 9 mths.	Ob.S/BS [%]	2.56 ± 0.64	4.36 ± 1.59	4.49 ± 1.44	1.67 ± 0.38	1.91 ± 0.38	**2.83 ± 0.52 #**
		OS/BS [%]	0.07 ± 0.07	3.81 ± 2.48	**9.47 ± 4.22 #**	0.07 ± 0.07	1.76 ± 1.18	1.91 ± 1.22
	50 µg 6 mths.	MS/BS [x-fold]	1.00 ± 0.00	1.09 ± 0.21	0.97 ± 0.16	n. a.	n. a.	n. a.
		MAR [x-fold]	1.00 ± 0.00	1.20 ± 0.11 *	**1.84 ± 0.34 #**	n. a.	n. a.	n. a.
		BFR/BS [x-fold]	1.00 ± 0.00	1.17 ± 0.28	1.42 ± 0.22	n. a.	n. a.	n. a.

HA = hydroxyapatite; BMP = bone morphogenetic protein; GDF = growth and differentiation factor; osteoblast surface (Ob.S/BS); osteoid surface (OS/BS); mineralized surface (MS/BS); mineral apposition rate (MAR), and bone formation rate (BFR/BS); n. a. = not applicable; values are means ± standard errors of the mean; * *p* ≤ 0.05 HA vs. control; # *p* ≤ 0.05 HA/BMP vs. control; + *p* ≤ 0.05 HA/BMP vs. HA; & *p* ≤ 0.05, && *p* ≤ 0.01 for 9 months or 6 months vs. 3 months. Indicated in bold: Data with significance values of *p* ≤ 0.05 for the explorative analysis with the non-parametric multigroup Kruskal-Wallis and two-sided Wilcoxon tests for the comparison of paired samples within one group, and the multigroup Friedman and two-sided Mann-Whitney U-tests for statistical evaluation of differences between groups.

## Data Availability

The data presented in this study are available on request from the corresponding author.

## Supplementary Materials

The following supporting information can be downloaded at: https://www.mdpi.com/article/10.3390/biomedicines10020513/s1, Supplementary data; Figure S1: Merz counting reticule with 36 hits (inflection points of the sinus curves) used for static histomorphometry.; Figure S2: Relative increase of bone mineral density (BMD) in lumbar vertebral bodies [L3 = control (100%), L4 = HA/BMP; L5 = HA] of groups injected with BMP-2 (A) or GDF-5 (B); Figure S3: Depiction of one representative of several islets of hyaline cartilage as a sign of early enchondral ossification in paraffin sections of lumbar vertebral bodies at 3 months after injection of HA particles; Table S1: Differences among the release of different doses of BMPs from the HA particles (BMP-2; GDF-5; *n* = triplicates each); Table S2: Results of the linear mixed effects model for treatment (group), time point (time), BMP dose (dose), and area-related effects of BMP-2 or GDF-5. References [54,57,91,92,93] are cited in the supplementary materials
