# Supplementary material for: Single Application of Low-Dose, Hydroxyapatite-Bound BMP-2 or GDF-5 Induces Long-Term Bone Formation and Biomechanical Stabilization of a Bone Defect in a Senile Sheep Lumbar Osteopenia Model
Source: Biomedicines. 2022 Feb 21;10(2):513. doi: 10.3390/biomedicines10020513 (PMC8962316; doi:10.3390/biomedicines10020513)
Supplement: Supplementary file 1 [file biomedicines-10-00513-s001.zip › Suppl Fig S1.pdf]

For static histomorphometry, the following values were determined in 25 fields in the immediate vicinity of the injection channel or the central remote area of each individual vertebral body using a standard microscope (Axiovert 200 M, Carl Zeiss Microimaging GmbH, Oberkochen, Germany), 400-fold magnification, and a Merz counting reticule (Supplementary Fig. S1; [1, 2]):

The following values were determined:

F	=	Number of measuring fields
P	=	Number of hits (points) over mineralized bone and osteoid
I	=	Total intersection number of the wave lines with the trabecular bone surface
I_{OB}	=	Intersections of the wave lines contacting osteoid with osteoblasts
I_{IO}	=	Intersections of the wave lines contacting osteoid without osteoblasts
d	=	distance between 2 adjacent points in the counting net

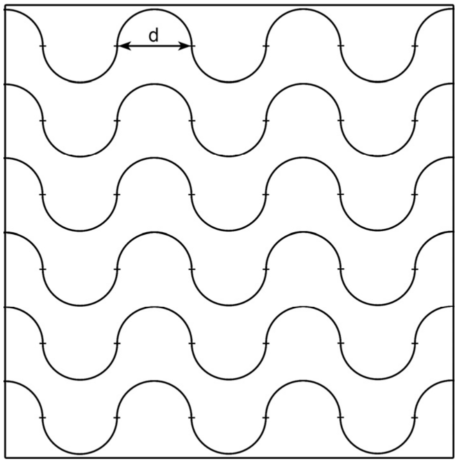

Supplementary Figure S1. Merz counting reticule with 36 hits (inflection points of the sinus curves) used for static histomorphometry.

The six parameters calculated below correspond to the recommendations of the International Committee of the American Society for Bone and Mineral Research (ASBMR) [3-5]:

Bone Volume (BV/TV): mineralized and non-mineralized fraction of the bone (includes the entire bone with cavities and appendix tissue)

$$BV/TV = P \times 100 / (F \times 36) \quad [\%] \quad (1)$$

Trabecular thickness (Tb.Th): average absolute thickness of the trabeculae

$$Tb.Th = 2 \times P \times d / I \quad [\mu m] \quad (2)$$

Cortical thickness (Ct.Th): average absolute thickness of the corticalis in the area of interest (ventral and lateral right corticalis of the vertebrae) [µm] (3)

Osteoblast surface (Ob.S/BS): surface proportion of the osteoblast-covered osteoid related to the total trabecular bone surface

$$Obs./BS = I_{OB} \times 100 / I \quad [\%] \quad (4)$$

Osteoid surface (OS/BS): non-mineralized surface fraction related to the total bone surface independent of the osteoblast coverage

$$OS/BS = (I_{OB} + I_{IO}) \times 100 / I \quad [\%] \quad (5)$$

Eroded Surface (ES/BS): surface proportion of resorption lacunae related to the total trabecular bone surface independent of the osteoclast coverage

$$ES/BS = (IHO + IHE) \times 100 / I \quad [\%] \quad (6)$$

Dynamic histomorphometric measurements (n=38 sheep) were performed by examining 10 randomly selected visual fields in the immediate vicinity of the injection channel in every sample with respect to OTC-single or double lines. The total bone surface (BS) and the mineralized fraction of the BS labeled with single or double line OTC were determined (MS) and the results expressed as the mineralized fraction (%; see below). In addition, the average distance between OTC double lines was determined

over their total length (Ir.L.TH = Interlabel thickness) and divided by the number of days between the subsequent injections of OTC (Ir.l.t. = Interlabel time; see below). The fluorescence values in the control vertebrae L3 were individually normalized to 1.0 (100%) and the results for L4 (BMP-coated HA-particles) and L5 (HA-particles) then expressed as fold-increase versus L3.

Mineralizing surface per bone surface (MS/BS):

MS/BS = (dLs + sLs/2)/BS x 100 [%] double labels (dLs) + ½ single labels (sLs)

Mineral apposition rate (MAR):

MAR = Ir.L.TH/Ir.l.t. [µm/d] Interlabel thickness/Interlabel time

Bone formation rate (BFR):

BFR = MS/BS x MAR [µm/d]

References

1. W.A. Merz, Die Streckenmessung an gerichteten Strukturen im Mikroskop und ihre Anwendung zur Bestimmung von Oberflächen-Volumen-Relationen im Knochengewebe, Mikroskopie 22 (1967) 132–142.

2. G. Delling, Endokrine Osteopathien, Fischer, Stuttgart, 1975.

3. B.A. Ashton, F. Kuhlencordt, L. Mohr, G. Bergmann, Handbuch der inneren Medizin, Springer, Berlin, 1980.

4. A. Parfitt, M. Drezner, F. Glorieux, J. Kanis, H. Malluche, P. Meunier, S. Ott, R. Recker, Bone histomorphometry: standardization of nomenclature, symbols and units, J. Bone Miner. Res. 2 (1987) 595–610.

5. D.W. Dempster, J.E. Compston, M.K. Drezner, F.H. Glorieux, J.A. Kanis, H. Malluche, P.J. Meunier, S.M. Ott, R.R. Recker, A.M. Parfitt, Standardized nomenclature, symbols, and units for bone histomorphometry: A 2012 update of the report of the ASBMR Histomorphometry Nomenclature Committee, J Bone. Miner. Re.s 28 (2013) 2–17.
