# Supplementary material for: Single Application of Low-Dose, Hydroxyapatite-Bound BMP-2 or GDF-5 Induces Long-Term Bone Formation and Biomechanical Stabilization of a Bone Defect in a Senile Sheep Lumbar Osteopenia Model
Source: Biomedicines. 2022 Feb 21;10(2):513. doi: 10.3390/biomedicines10020513 (PMC8962316; doi:10.3390/biomedicines10020513)
Supplement: Supplementary file 1 [file biomedicines-10-00513-s001.zip › Suppl Fig S2_S3.pptx]

## Slide 1
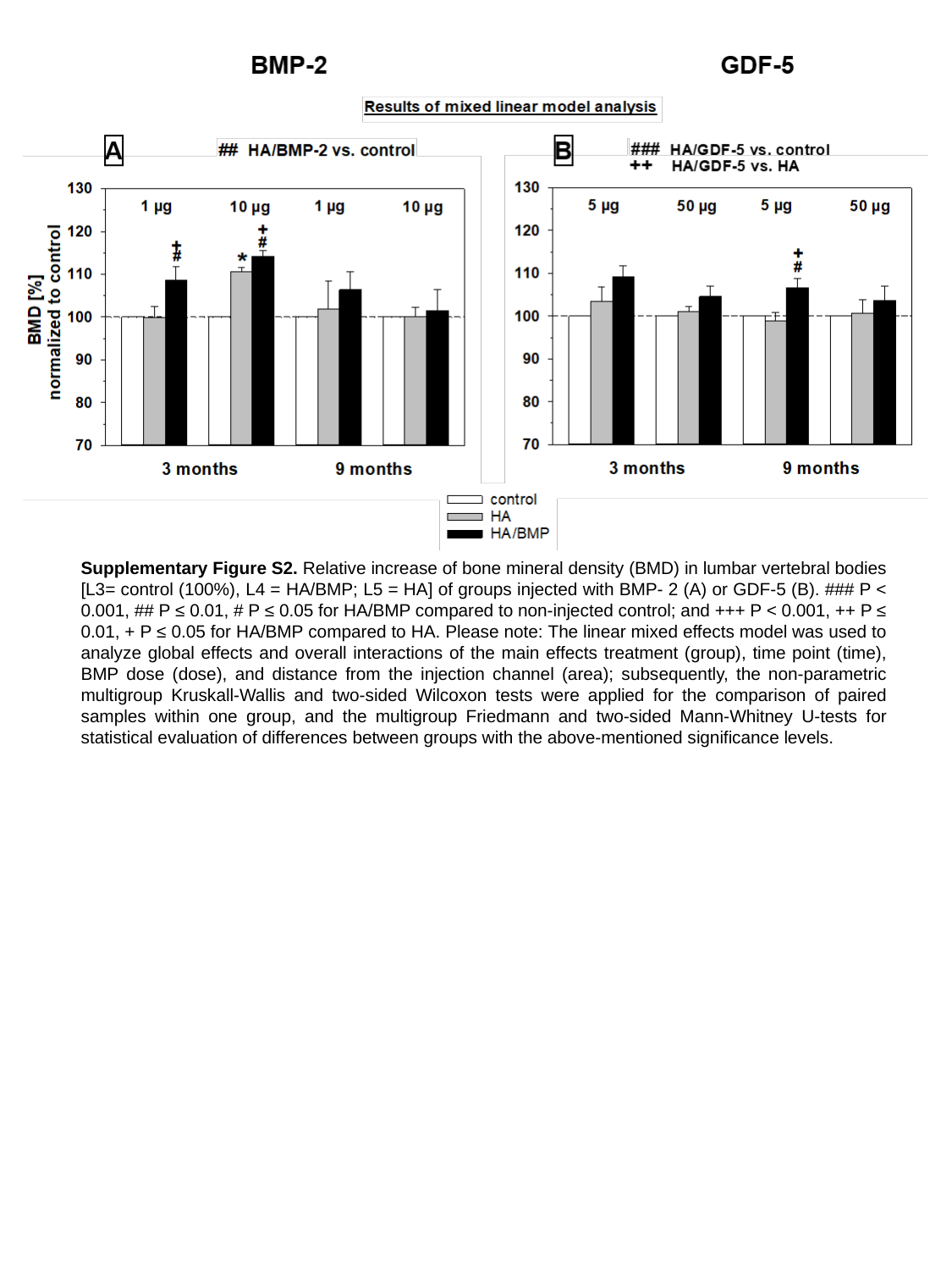

Supplementary Figure S2. Relative increase of bone mineral density (BMD) in lumbar vertebral bodies [L3= control (100%), L4 = HA/BMP; L5 = HA] of groups injected with BMP- 2 (A) or GDF-5 (B). ### P < 0.001, ## P ≤ 0.01, # P ≤ 0.05 for HA/BMP compared to non-injected control; and +++ P < 0.001, ++ P ≤ 0.01, + P ≤ 0.05 for HA/BMP compared to HA. Please note: The linear mixed effects model was used to analyze global effects and overall interactions of the main effects treatment (group), time point (time), BMP dose (dose), and distance from the injection channel (area); subsequently, the non-parametric multigroup Kruskall-Wallis and two-sided Wilcoxon tests were applied for the comparison of paired samples within one group, and the multigroup Friedmann and two-sided Mann-Whitney U-tests for statistical evaluation of differences between groups with the above-mentioned significance levels.

## Slide 2
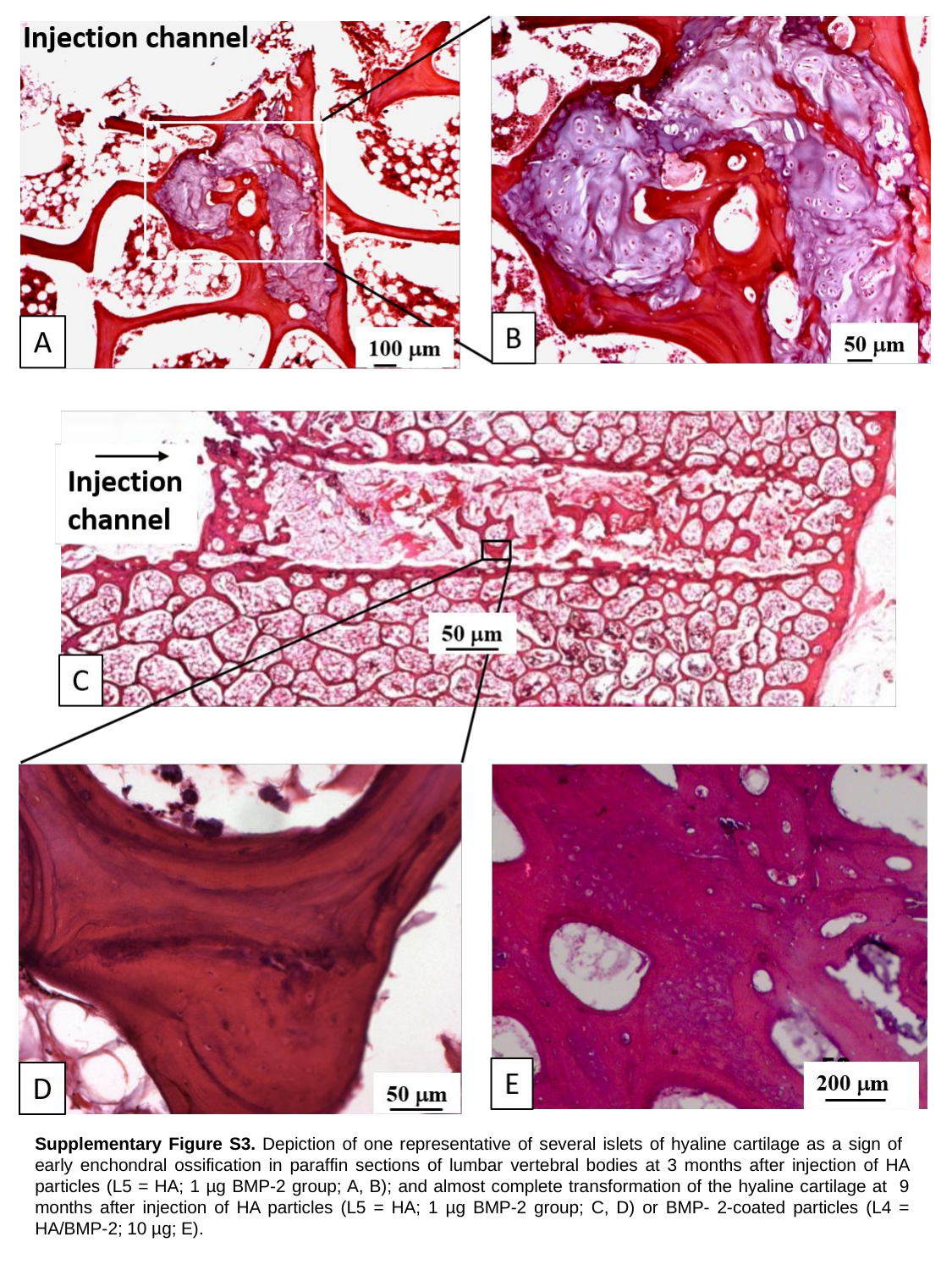

Supplementary Figure S3. Depiction of one representative of several islets of hyaline cartilage as a sign of early enchondral ossification in paraffin sections of lumbar vertebral bodies at 3 months after injection of HA particles (L5 = HA; 1 µg BMP-2 group; A, B); and almost complete transformation of the hyaline cartilage at 9 months after injection of HA particles (L5 = HA; 1 µg BMP-2 group; C, D) or BMP- 2-coated particles (L4 = HA/BMP-2; 10 µg; E).
