# Supplementary material for: Single Application of Low-Dose, Hydroxyapatite-Bound BMP-2 or GDF-5 Induces Long-Term Bone Formation and Biomechanical Stabilization of a Bone Defect in a Senile Sheep Lumbar Osteopenia Model
Source: Biomedicines. 2022 Feb 21;10(2):513. doi: 10.3390/biomedicines10020513 (PMC8962316; doi:10.3390/biomedicines10020513)
Supplement: Supplementary file 1 [file biomedicines-10-00513-s001.zip › Supplementary_Table_1.pdf]

Supplementary Table S1: Differences among the release of different doses of BMPs from the HA particles (BMP-2; GDF-5; n = triplicates each).

		BMP-2 (2 µg)											GDF-5 (10 µg)										
Time point (days)		0.5 h	1 h	4 h	6 h	1	2	4	7	9	11	14	0.5 h	1 h	4 h	6 h	1	2	4	7	9	11	14
PBS	Release/d (ng/ml)	0.36	0.40	0.28	0.31	0.31	0.38	0.43	0.42	0.41	0.37	0.37	8.63	13.32	18.95	12.71	14.83	20.05	14.10	9.82	8.68	5.75	7.05
PBS	Cumulative Release (ng)	0.36	0.76	1.03	1.34	1.65	2.03	2.46	2.88	3.30	3.66	**4.03**	8.63	21.95	40.90	53.61	68.44	88.49	102.6	112.4	121.1	126.8	**133.9**
PBS	Release (%)	0.02	0.04	0.05	0.07	0.08	0.10	0.12	0.14	0.16	0.18	0.20	0.09	0.22	0.41	0.54	0.68	0.88	1.03	1.12	1.21	1.27	1.34
PBS	Retention (%)	100.0	100.0	99.9	99.9	99.9	99.9	99.9	99.9	99.8	99.8	99.8	99.91	99.78	99.59	99.46	99.32	99.12	98.97	98.88	98.79	98.73	98.66
		BMP-2 (20 µg)											GDF-5 (100 µg)										
PBS	Release/d (ng/ml)	0.85	0.91	0.91	0.92	1.02	0.90	0.87	0.75	0.92	1.04	0.96	22.95	33.54	33.41	32.22	40.13	56.24	36.04	43.18	35.35	24.74	28.86
PBS	Cumulative Release (ng)	0.85	1.76	2.67	3.58	4.60	5.50	6.37	7.12	8.04	9.08	**10.04**	22.95	56.49	89.90	122.1	162.3	218.5	254.5	297.7	333.1	357.8	**386.7**
PBS	Release (%)	0.00	0.01	0.01	0.02	0.02	0.03	0.03	0.04	0.04	0.05	0.05	0.02	0.06	0.09	0.12	0.16	0.22	0.25	0.30	0.33	0.36	0.39
PBS	Retention (%)	100.0	99.99	99.99	99.98	99.98	99.97	99.97	99.96	99.96	99.95	99.95	99.98	99.94	99.91	99.88	99.84	99.78	99.75	99.70	99.67	99.64	99.61
		BMP-2 (2 µg)											GDF-5 (10 µg)										
Time point (days)		0.5 h	1 h	4 h	6 h	1	2	4	7	9	11	14	0.5 h	1 h	4 h	6 h	1	2	4	7	9	11	14
Serum	Release (ng/ml)	720.7	335.0	213.9	107.9	101.8	40.03	26.52	15.99	8.70	6.50	4.88	4423	1572	1106	580.6	431.7	251.9	136.6	99.60	30.39	19.97	16.72
Serum	Cumulative Release (ng)	720.7	1056	1270	1377	1479	1519	1546	1562	1570	1577	**1582**	4423	5995	7101	7682	8113	8365	8502	8601	8632	8652	**8668**
Serum	Release (%)	36.03	52.78	63.48	68.87	73.96	75.96	77.28	78.08	78.52	78.84	79.09	44.23	59.95	71.01	76.82	81.13	83.65	85.02	86.01	86.32	86.52	86.68
Serum	Retention (%)	63.96	47.22	36.52	31.13	26.04	24.04	22.72	21.92	21.48	21.16	20.91	55.77	40.05	28.99	23.18	18.87	16.35	14.98	13.99	13.68	13.48	13.32
		BMP-2 (20 µg)											GDF-5 (100 µg)										
Serum	Release (ng/ml)	4674	4494	3841	2103	2391	1097	751.7	496.9	146.8	128.3	80.74	11431	6389	10353	4986	8631	8444	7127	6049	5134	4121	3426
Serum	Cumulative Release (ng)	4674	9168	13009	15112	17503	18600	19352	19849	19995	20124	**20204**	11326	19560	28173	33159	41790	50234	57361	63410	68544	72665	**76092**
Serum	Release (%)	23.37	45.84	65.05	75.56	87.52	93.00	96.76	99.24	99.98	100	100	11.43	17.82	28.17	33.16	41.79	50.23	57.36	63.41	68.54	72.67	76.09
Serum	Retention (%)	76.63	54.16	34.95	24.44	12.48	7.00	3.24	0.76	0.02	0.00	0.00	88.57	82.18	71.83	66.84	58.21	49.77	42.64	36.59	31.46	27.33	23.91
