# Supplementary material for: Single Application of Low-Dose, Hydroxyapatite-Bound BMP-2 or GDF-5 Induces Long-Term Bone Formation and Biomechanical Stabilization of a Bone Defect in a Senile Sheep Lumbar Osteopenia Model
Source: Biomedicines. 2022 Feb 21;10(2):513. doi: 10.3390/biomedicines10020513 (PMC8962316; doi:10.3390/biomedicines10020513)
Supplement: Supplementary file 1 [file biomedicines-10-00513-s001.zip › Supplementary_Table_2_GLM_BMP2_16_02_22_mit_CI_Version_2.pdf]

Supplementary Table S2. Results of the linear mixed effects model for treatment (group), time point (time), BMP dose (dose), and area-related effects of BMP-2 or GDF-5.

Parameter		Treatment (adjacent area 1)			Treatment (remote area 2)			group	time	dose	area	group*time	group*dose	group * area
		HA vs. control	HA-BMP-2 vs. control	HA-BMP-2 vs. HA	HA vs. control	HA-BMP-2 vs. control	HA-BMP-2 vs. HA							
**BMD [%]** (area 1)	P (η_p^2)	0.318	**0.001**	0.069	n.a.	n.a.	n.a.	**0.001** (0.285)	0.085 (0.135)	0.463 (0.026)	n.a.	0.154 (0.085)	0.437 (0.039)	n.a.
	Est. Δ vs. Ctr./HA	3.162	7.678	4.515	n.a.	n.a.	n.a.		-3.883	1.605	n.a.			
	Confidence interval (95%)	-1.605 to 7.929	2.911 to 12.444	-0.252 to 9.282	n.a.	n.a.	n.a.		-8.346 to 0.579	-2.858 to 6.067	n.a.			
**BV/TV [%]**	P (η_p^2)	**< 0.001**	**< 0.001**	**< 0.001**	**< 0.001**	**< 0.001**	**0.032**	**< 0.001** (0.749)	0.081 (0.152)	0.184 (0.091)	**< 0.001** (0.551)	0.745 (0.006)	0.435 (0.016)	**< 0.001** (0.375)
	Est. Δ vs. Ctr./HA	15.202	27.011	11.809	3.300	10.760	7.460		-3.076	-2.311	-9.810			
	Confidence interval (95%)	12.199 to 18.205	24.008 to 30.014	8.806 to 14.812	0.297 to 6.304	7.757 to 13.763	4.456 to 10.463		-6.567 to 0.415	-5.816 to 1.194	0.076 to -11.544			
**Tb.Th [µm]**	P (η_p^2)	**< 0.001**	**< 0.001**	**< 0.001**	0.128	**< 0.001**	**< 0.001**	**< 0.001** (0.730)	0.335 (0.049)	0.069 (0.163)	**< 0.001** (0.629)	0.494 (0.014)	**0.036** (0.064)	**< 0.001** (0.442)
	Est. Δ vs. Ctr./HA	106.277	213.938	107.661	18.417	65.571	47.101		-15.266	-29.807	-89.039			
	Confidence interval (95%)	82.413 to 130.140	190.075 to 237.802	83.798 to 130.140	-5.393 to 42.334	41.708 to 89.435	23.237 to 70964		-47.546 to 17.013	-62.221 to 2.607	-75.261 to -102.816			
**Cort. Th [µm]**	P (η_p^2)	0.054	**0.002**	0.243	**0.014**	**0.007**	0.795	**< 0.001** (0.160)	0.185 (0.090)	**0.011** (0.291)	**0.001** (0.114)	0.231 (0.029)	0.631 (0.009)	0.830 (0.004)
	Est. Δ vs. Ctr./HA	103.586	165.137	61.551	131.600	145.478	13.878		93.901	192.360	-107.773			
	Confidence interval (95%)	-1.869 to 209.041	59.682 to 270.592	-42.482 to 165.584	27.567 to 235.633	40.023 to 250.933	-91.577 to 119.333		-49.025 to 236.827	48.871 to 335.849	-168.387 to -47.159			
**Ob.S/BS [%]**	P (η_p^2)	**0.004**	**< 0.001**	0.141	0.466	0.051	0.215	**< 0.001** (0.164)	**0.006** (0.340)	**0.047** (0.192)	0.906 (0.000)	0.115 (0.042)	0.122 (0.041)	0.149 (0.037)
	Est. Δ vs. Ctr./HA	0.739	1.108	0.369	0.182	0.493	0.310		1.193	-0.814	-0.017			
	Confidence interval (95%)	0.245 to 1.233	0.614 to 1.602	-0.125 to 0.863	-0.312 to 0.676	-0.001 to 0.987	-0.183 to 0.804		0.395 to 1.991	-1.615 to -0.012	-0.302 to 0.268			
**OS/BS [%]**	P (η_p^2)	**0.014**	**0.001**	0.303	0.509	0.071	0.509	**0.002** (0.137)	0.212 (0.096)	0.131 (0.136)	**0.045** (0.046)	0.597 (0.012)	0.433 (0.019)	0.357 (0.024)
	Est. Δ vs. Ctr./HA	7.947	11.236	3.288	2.104	5.795	3.691		-5.565	-6.802	-3.761			
	Confidence interval (95%)	1.641 to 14.253	4.930 to 17.541	-3.017 to 9.594	-4.201 to 8.410	-0.511 to 12.101	-2.615 to 9.996		-14.635 to 3.504	-15.871 to 2.268	-7.402 to -0.121			
**MS/BS [X]** (area 1)	P (η_p^2)	0.083	1.000	**0.017**	n.a.	n.a.	n.a.	**0.015** (0.260)	0.746 (0.008)	0.283 (0.082)	n.a.	0.507 (0.047)	0.289 (0.085)	n.a.
	Est. Δ vs. Ctr./HA	-3.668	1.051	4.718	n.a.	n.a.	n.a.		1.322	-4.470	n.a.			
	Confidence interval (95%)	-7.689 to 0.353	-2.970 to 5.071	0.698 to 8.739	n.a.	n.a.	n.a.		-7.262 to 9.906	-13.054 to 4.114	n.a.			
**MAR [X]** (area 1)	P (η_p^2)	**0.043**	1.000	**0.014**	n.a.	n.a.	n.a.	**0.015** (0.259)	**0.038** (0.273)	0.220 (0.105)	n.a.	0.926 (0.005)	0.814 (0.015)	n.a.
	Est. Δ vs. Ctr./HA	-2.471	0.417	2.888	n.a.	n.a.	n.a.		3.431	-1.924	n.a.			
	Confidence interval (95%)	-4.879 to -0.063	-1.991 to 2.825	0.480 to 5.296	n.a.	n.a.	n.a.		0.219 to 6.643	-5.163 to 1.288	n.a.			
**BFR/BS [X]** (area 1)	P (η_p^2)	**0.030**	1.000	**0.019**	n.a.	n.a.	n.a.	**0.012** (0.270)	0.064 (0.224)	0.107 (0.175)	n.a.	0.732 (0.022)	0.219 (0.103)	n.a.
	Est. Δ vs. Ctr./HA	-0.540	0.036	0.576	n.a.	n.a.	n.a.		0.923	-0.791	n.a.			
	Confidence interval (95%)	-1.039 to -0.041	-0.463 to 0.535	0.077 to 1.075	n.a.	n.a.	n.a.		-0.062 to 1.908	-1.776 to 0.195	n.a.			
**ES/BS [%]**	P (η_p^2)	0.251	0.354	0.823	0.833	0.436	0.569	0.774 (0.006)	**0.030** (0.262)	0.528 (0.025)	0.556 (0.004)	0.663 (0.010)	**0.038** (0.073)	0.442 (0.019)
	Est. Δ vs. Ctr./HA	0.401	0.323	-0.078	-0.074	-0.272	-0.198		-0.707	0.191	0.118			
	Confidence interval (95%)	-0.289 to 1.091	-0.367 to 1.013	-0.768 to 0.612	-0.764 to 0.616	-0.962 to 0.418	-0.888 to 0.492		-1.336 to -0.078	-0.438 to 0.820	-0.280 to 0.516			

Parameter		Treatment (adjacent area 1)			Treatment (remote area 2)			group	time	dose	area	group*time	group*dose	group * area
		HA vs. control	HA-GDF-5 vs. control	HA-GDF-5 vs. HA	HA vs. control	HA-GDF-5 vs. control	HA-GDF-5 vs. HA							
**BMD [%]** (area 1)	P (η_p^2)	1.000	**< 0.001**	**0.003**	n.a.	n.a.	n.a.	**< 0.001** (0.332)	0.302 (0.051)	0.315 (0.048)	n.a.	0.672 (0.019)	0.327 (0.052)	n.a.
	Est. Δ vs. Ctr./HA	1.045	6.011	4.966	n.a.	n.a.	n.a.		-1.406	-1.368	n.a.			
	Confidence interval (95%)	-2.412 to 4.503	2.554 to 9.468	1.509 to 8.423	n.a.	n.a.	n.a.		-4.168 to 1.355	-4.129 to 1.394	n.a.			
**BV/TV [%]**	P (η_p^2)	**< 0.001**	**< 0.001**	**< 0.001**	**< 0.001**	**< 0.001**	**0.032**	**< 0.001** (0.805)	**< 0.001** (0.662)	0.185 (0.090)	**< 0.001** (0.754)	0.615 (0.010)	0.113 (0.042)	**< 0.001** (0.624)
	Est. Δ vs. Ctr./HA	16.251	30.968	14.717	0.711	7.325	6.614		5.633	-1.274	-13.347			
	Confidence interval (95%)	13.629 to 18.873	28.346 to 33.590	12.096 to 17.339	-1.911 to 3.333	4.703 to 9.947	3.992 to 9.236		3.701 to 7.566	-3.214 to 0.667	-14.861 to -11.834			
**Tb.Th [µm]**	P (η_p^2)	**< 0.001**	**< 0.001**	**< 0.001**	0.771	**0.003**	**0.007**	**< 0.001** (0.664)	**< 0.001** (0.633)	0.404 (0.037)	**< 0.001** (0.625)	**0.013** (0.082)	0.298 (0.024)	**< 0.001** (0.486)
	Est. Δ vs. Ctr./HA	114.489	251.060	136.571	4.479	46.840	42.361		64.086	-9.597	-110.937			
	Confidence interval (95%)	84.108 to 144.879	220.679 to 281.441	106.190 to 166.952	-25.902 to 34.860	16.459 to 77.221	11.980 to 72.742		40.660 to 87.512	-33.120 to 13.927	-128.478 to -93.369			
**Cort. Th [µm]**	P (η_p^2)	**0.001**	**< 0.001**	**< 0.001**	0.238	**< 0.001**	**0.007**	**< 0.001** (0.363)	0.613 (0.014)	0.383 (0.040)	**< 0.001** (0.199)	0.520 (0.013)	0.222 (0.029)	0.131 (0.039)
	Est. Δ vs. Ctr./HA	121.545	243.133	121.587	43.280	143.407	100.127		24.924	43.456	-105.446			
	Confidence interval (95%)	49.171 to 193.920	170.758 to 315.507	49.213 to 193.920	-29.095 to 115.655	71.033 to 215.782	27.753 to 172.502		-76.577 to 126.425	-58.467 to 145.378	-147.232 to -63.661			
**Ob.S/BS [%]**	P (η_p^2)	0.070	**0.003**	0.250	0.808	0.106	0.168	**0.006** (0.097)	**0.001** (0.420)	0.530 (0.021)	**< 0.001** (0.189)	0.116 (0.042)	0.651 (0.008)	0.477 (0.015)
	Est. Δ vs. Ctr./HA	0.631	1.030	0.399	0.084	0.562	0.478		1.557	0.270	-0.959			
	Confidence interval (95%)	-0.052 to 1.315	0.347 to 1.713	-0.285 to 1.082	-0.599 to 0.767	-0.121 to 1.245	-0.205 to 1.161		0.679 to 2.436	-0.612 to 1.151	-1.354 to -0.565			
**OS/BS [%]**	P (η_p^2)	**< 0.001**	**< 0.001**	0.178	0.283	0.105	0.579	**< 0.001** (0.274)	**< 0.001** (0.763)	0.227 (0.076)	**< 0.001** (0.188)	**0.005** (0.098)	0.728 (0.006)	**0.003** (0.107)
	Est. Δ vs. Ctr./HA	11.965	15.397	3.431	2.733	4.141	1.408		-12.956	-2.060	-6.830			
	Confidence interval (95%)	6.948 to 16.983	10.379 to 20.414	-1.586 to 8.449	-2.285 to 7.750	-0.877 to 9.158	-3.609 to 6.426		-16.421 to -9.490	-5.511 to 1.391	-9.726 to -3.933			
**MS/BS [X]** (area 1)	P (η_p^2)	1.000	1.000	1.000	n.a.	n.a.	n.a.	0.629 (0.025)	**< 0.001** (0.679)	0.577 (0.018)	n.a.	0.668 (0.022)	0.293 (0.066)	n.a.
	Est. Δ vs. Ctr./HA	0.228	0.703	0.475	n.a.	n.a.	n.a.		11.331	1.033	n.a.			
	Confidence interval (95%)	-1.847 to 2.303	-1.373 to 2.778	-1.601 to 2.550	n.a.	n.a.	n.a.		7.471 to 15.192	-2.792 to 4.859	n.a.			
**MAR [X]** (area 1)	P (η_p^2)	1.000	**< 0.001**	**< 0.001**	n.a.	n.a.	n.a.	**< 0.001** (0.310)	**0.001** (0.194)	**0.047** (0.071)	n.a.	0.643 (0.016)	0.061 (0.098)	n.a.
	Est. Δ vs. Ctr./HA	0.069	2.729	2.660	n.a.	n.a.	n.a.		1.859	1.038	n.a.			
	Confidence interval (95%)	-1.508 to 1.647	1.152 to 4.307	1.083 to 4.238	n.a.	n.a.	n.a.		0.801 to 2.918	-0.010 to 2.087	n.a.			
**BFR/BS [X]** (area 1)	P (η_p^2)	1.000	**0.003**	**0.002**	n.a.	n.a.	n.a.	**0.002** (0.298)	**< 0.001** (0.602)	0.194 (0.092)	n.a.	0.776 (0.014)	0.435 (0.045)	n.a.
	Est. Δ vs. Ctr./HA	-0.002	0.246	0.248	n.a.	n.a.	n.a.		0.507	0.130	n.a.			
	Confidence interval (95%)	-0.173 to 0.169	0.075 to 0.417	0.077 to 0.419	n.a.	n.a.	n.a.		0.303 to 0.711	-0.072 to 0.332	n.a.			
**ES/BS [%]**	P (η_p^2)	0.424	0.338	0.873	0.562	0.377	0.145	0.514 (0.013)	**0.001** (0.472)	0.637 (0.012)	0.420 (0.006)	0.572 (0.011)	0.420 (0.017)	0.368 (0.020)
	Est. Δ vs. Ctr./HA	-0.229	-0.275	-0.046	-0.166	0.254	0.420		-0.979	-0.113	0.134			
	Confidence interval (95%)	-0.796 to 0.337	-0.841 to 0.292	-0.612 to 0.521	-0.733 to 0.400	-0.313 to 0.820	-0.147 to 0.986		-1.476 to -0.482	-0.608 to 0.381	-0.193 to 0.461			

Bone mineral density (BMD); bone volume/total volume (BV/TV); trabecular thickness (Tb.Th); osteoblast surface (Ob.S/BS); osteoid surface (OS/BS); cortical thickness (Cort. Th); mineralizing surface per bone surface (MS/BS); mineral apposition rate (MAR); bone formation rate surface-based BFR/BS; and eroded surface (ES/BS); η_p^2 = partial eta squared; Est. Δ vs. Ctr./HA = estimate Δ compared to control/HA; HA = hydroxyapatite particles; BMP-2 = bone morphogenetic protein-2; GDF-5 = growth/differentiation factor-5; n.a. = not applicable; a linear mixed effects model was fitted to compare the effects of treatment (group), time point after treatment (time), BMP dose (dose), and distance from the injection channel (area), as well as the interactions (*) between group on one hand and the parameters time, dose, and area on the other hand, on the nine different static and dynamic histomorphometrical parameters. In this analysis, group, time, dose, and area were modeled as fixed effects with a random intercept per sheep, in order to account for multiple measurements per sheep and the correlation of these observations. η_p^2 from the model was used to assess the effect size (classification: $0.01 \leq \eta_p^2 < 0.06$ – small effect size; $0.06 \leq \eta_p^2 < 0.14$ – medium effect size; $\eta_p^2 \geq 0.14$ – large effect size; color coding applied to columns 9 to 15). The same color coding is applied in columns 3 to 8 for P ≤ 0.05; ≤ 0.01; < 0.001.
